# Epigenetic targeting drugs potentiate chemotherapeutic effects in solid tumor therapy

**DOI:** 10.1038/s41598-017-04406-0

**Published:** 2017-06-22

**Authors:** Jingjing Li, Dapeng Hao, Li Wang, Haitao Wang, Yuan Wang, Zhiqiang Zhao, Peipei Li, Chuxia Deng, Li-jun Di

**Affiliations:** 1 Cancer Center, Faculty of Health Sciences, University of Macau, Macau, China; 2 Metabolomics Core, Faculty of Health Sciences, University of Macau, Macau, China

## Abstract

Epigenetic therapy is a novel tumor therapeutic method and refers to the targeting of the aberrant epigenetic modifications presumably at cancer-related genes by chemicals which are epigenetic targeting drugs (ETDs). Not like in treating hematopoietic cancer, the clinical trials investigating the potential use of ETDs in the solid tumor is not encouraging. Instead, the curative effects of ETD delivered together with DNA targeting chemo drugs (DTDs) are quite promising according to our meta-analysis. To investigate the synergistic mechanism of ETD and DTD drug combination, the therapeutic effect was studied using both cell lines and mouse engrafted tumors. Mechanically we show that HDAC inhibitors and DNMT inhibitors are capable of increasing the chromatin accessibility to cisplatin (CP) and doxorubicin (Dox) through chromatin decompaction globally. Consequently, the combination of ETD and DTD enhances the DTD induced DNA damage and cell death. Engrafted tumors in SCID mice also show increased sensitivity to irradiation (IR) or CP when the tumors were pretreated by ETDs. Given the limited therapeutic effect of ETD alone, these results strongly suggest that the combination of DTD, including irradiation, and ETD treatment is a very promising choice in clinical solid tumor therapy.

## Introduction

Epigenetic refers to the DNA methylation, histone modifications and the dynamic binding of variable proteins that shapes the chromatin compaction status and therefore, determines the gene expression through locally regulating the accessibility of chromatin to transcriptional factors and the ability to form active transcriptional higher order chromatin organization^1, 2^. Although it is the combination of DNA methylation, histone modification and chromatin remodeling which forms the “epigenetic code” to indicate the chromatin status, generally highly compact chromatin is labeled by low histone acetylation while open chromatin is more likely to have high acetylated histones^3–5^. Similarly, hypermethylated DNA generally indicates the chromatin silencing and DNA hypomethylation is more common at active transcribing chromatin region^6^.

Global alteration of epigenetic modification is considered as one of the hallmarks of cancer^7^. Hyperacetylation of histones and hypomethylation of DNA are both known to be overwhelmingly abundant in cancer cells^8, 9^. However, hypoacetylation of histones and hypermethylation of DNA at specific gene loci are also maintained^10–13^. Gene locus specific recruitment of epigenetic modifiers such as DNA methyl transferases (DNMT) and histone deacetylases (HDACs) are known to be important in maintaining the modification status of these gene loci in cancer cells^14, 15^. These gene loci are frequently discovered to be tumor suppressor genes (TSGs), which raises an interesting supposition that epigenetic silencing of TSG is another important mechanism for cancer formation^13, 16^. In the perspective of cancer therapy, the silenced TSG becomes an attractive target because recovery of the expression of TSG is much easier than repairing a mutated TSG gene. The purpose, eventually, is to get the cancer cells back to the differentiation program^17–19^, because TSGs may be able to persuade the cancer cell to undergo differentiation rather than continuing cell cycling. Another possible consequence of reactivating TSG is to kill the cancer cells because the cancer cells may not be able to survive when the TSG is back.

Targeting the epigenetic aberrations in cancer is now known as “epigenetic therapy” which mainly relies on the several DNMT inhibitors and HDAC inhibitors. Some promising results have been obtained in treating some types of hematopoietic cancer such as MDS, multiple myeloma and some lymphoma etc.^7, 20–22^. However, the clinical trials in treating the solid tumors using epigenetic therapy are now turning out as controversial or no effect at all^23, 24^ and the mechanism is still not clear. For instance, the most recent approved HDAC inhibitor panobinostat for multiple myeloma has also been shown to be effective in treating solid tumors^25^. Comparing to “epigenetic therapy”, chemotherapy and radiotherapy are still the first line choices for cancer treatment immediately after surgical removal of the tumor mass. Generating significant amount of DNA damage and inducing the cell death are the main outcome of these therapeutic methods. However, the therapeutic efficiency is also negatively influenced by the resistant mechanisms such as exportation of drugs by membrane-bound exporters, degradation of drugs by drug metabolism enzymes and the counterbalance of the drugs by antioxidants etc.^26^. Also, the dosage of the chemotherapeutic drugs and the irradiation cannot exceed the threshold of the patients’ tolerance, which increases the chance of cancer cell survival and the development of new lineage of cancer cells that are resistant to the therapy.

The most heavily used chemo drug in the clinic is DNA binding chemicals such as CP and Dox^27, 28^. The efficiency of these drugs is also influenced by the chromatin status^29^. Opened chromatin incorporates these drugs more easily than compact chromatin. Similar correlation also applies to irradiation^30, 31^. In this report, we found that epigenetic therapy increases the accessibility of the traditional chemo drugs such as DTDs and significantly improves the therapeutic effect of DTD in both the cancer cells cultured *in vitro* and the mouse engrafted tumors *in vivo*. Our data provide an insightful mechanical explanation of the beneficial effects when cancer patients were treated by ETD and DTD and support the clinical application of ETD and DTD combination.

## Results

### Beneficial outcome of combined implications between ETD and DTD in solid tumor therapy

**Figure 1:**
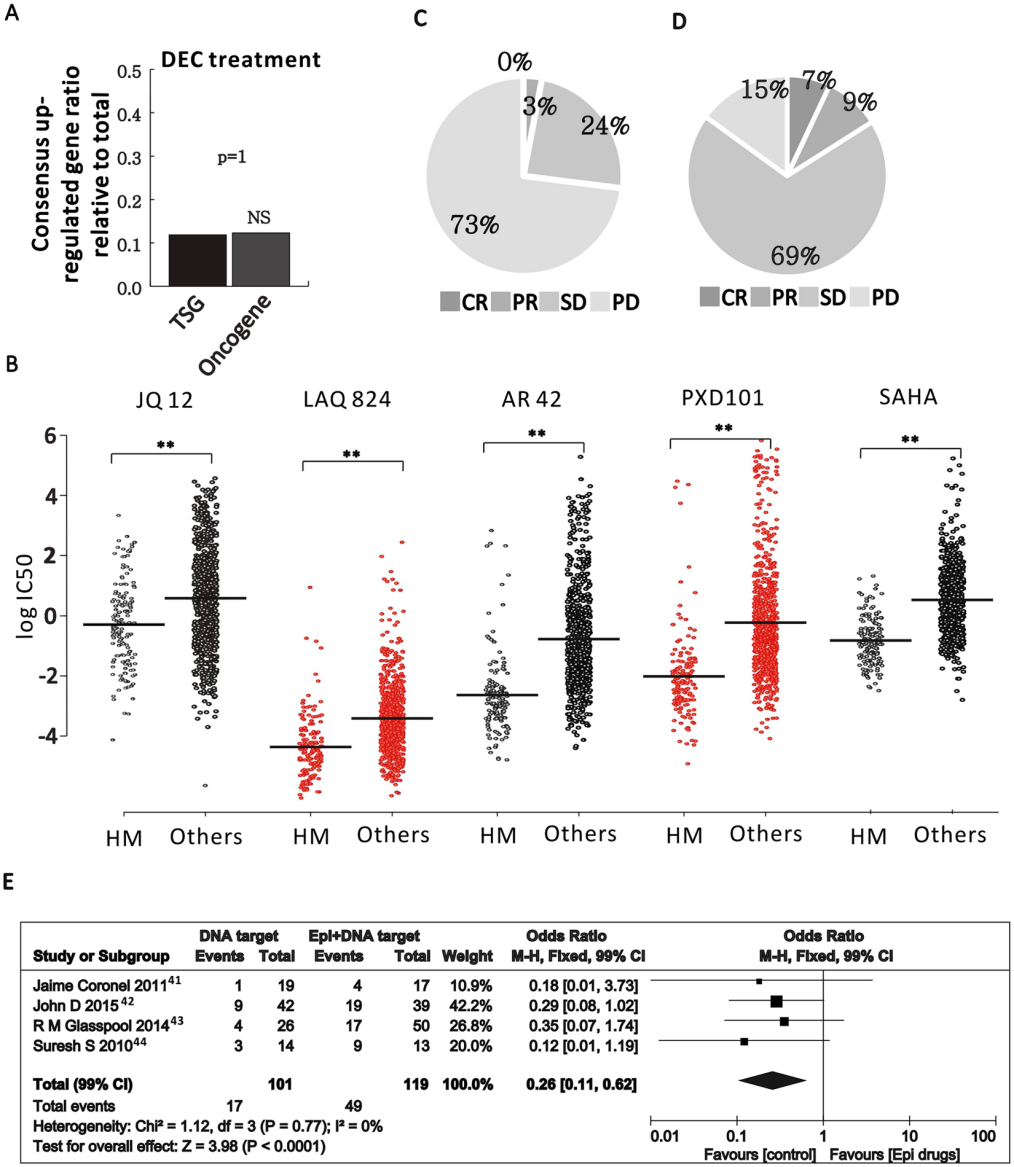
Epigenetic targeting drugs application in clinical. (**A**) Global gene expression from 18 primary AML samples in response to DEC treatment were collected (GSE40442) and the portion of consensus upregulated TSGs or Oncogenes, relative to total TSGs or oncogenes are plotted. P value is based on Chi-square test. (**B**) IC 50 of hematopoietic (HM) versus other cell lines to HDAC inhibitors. Each dot represents the IC 50 of one cell line to one drug as labeled on the top. The left group is the HM cell lines and the right group is the collection of all the other cell lines except HM cell lines. (**C**) The response rate of ETD alone applied to 1110 patients with solid tumors. CR, complete response; PR, partial response; SD, stable disease; PD, progressive disease. Response identification based on Response Evaluation Criteria in Solid Tumors (RECIST). (**D**) The response rate of classic chemical drugs applied to solid tumors according to TCGA. (**E**) Forest plot of objective response rate (ORR) related to DTD combined with ETD. The overall effect was statistically significant (P < 0.0001). Vertical line, “no-difference” point between two regimens; Horizontal line, 99% CI; Square, mean difference; Diamond, pooled mean difference for all studies. M-H fixed = Mantel-Haenszel fixed model. An odds ratio less than 1 means that combined therapy has successfully inhibited tumor progression.

Epigenetic markers becomes an attractive therapeutic concept in recent years and substantial progression has been made, especially in treating the hematopoietic malignancies^20, 25, 32–36^ (Table 1). Many of these epigenetic targeting chemicals were licensed to treat patients with defined cancer subtypes. For example, HDAC inhibitors including suberanilohydroxamic acid (SAHA), also known as Vorinostat, and Romidepsin (Rom), were mainly prescribed to treat some types of B cell lymphoma. Decitabine (DEC) is DNMT inhibitor and was licensed to treat myelodysplastic syndrome (MDS). A well-recognized explanation is that the tumor suppressor genes, which are repressed/silenced in cancer, can be reactivated by these drugs^23, 37^. However, the HDACi/DNMTi treatment of leukemia cells or non-malignant cells promoted global gene expression alteration which includes the up-regulation of not only tumor suppressors but also the oncogenes^38^ (Supplementary Fig. 1). Calculation of the gene number suggests no difference between the upregulated tumor suppressors and oncogenes (Fig. 1A). Instead, HDAC1 and DNMT1, the main known HDAC and DNMT factors in cancer have highest expression in hematopoietic malignancies, especially the B-cell lymphoma in the pan-cancer expression analysis (cBioportal, Supplementary Fig. 2) which may be the reason of the effectiveness of HDACi and DNMTi in treating these cancers. By analyzing the drug response of over 700 cell lines from different tissues, the hematopoietic cancer cell lines show obvious high response to several HDAC inhibitors (Fig. 1B), suggesting hematopoietic cells are more sensitive to this category of drugs specifically. Most of the solid tissue-related cancers show no obvious correlation to highly expressed HDACs and DNMTs, which is consistent with the poor outcome in 23 clinical trials exploring the potential of HDACi and DNMTi in treating solid tumor (Fig. 1C and Table 2) by comparing to the traditional chemotherapeutic drugs treatment (Fig. 1D)^39^.

**Table 1:** Epigenetic drugs approved by FDA.

Drugs	classification	Approved Year	Indicated disease	ORR
**Azacytidine** ^20^	DNMT inhibitor	2004	MDS	17.9%
**Vorinostat** ^32^	HDAC inhibitor	2006	CTCL	30%
**Decitabine** ^35^	DNMT inhibitor	2006	MDS	42%~54%
**Romidepesin** ^34^	HDAC inhibitor	2009	TCL	34%
**Ruxolitinib** ^36^	JAK1/2 inhibitor	2011	Myelofibrosis	30%
**Belinostat** ^33^	HDAC inhibitor	2015	PTCL	25.8%
**Panobinostat** ^25^	HDAC inhibitor	2015	MM	NA

MDS, Myelodysplastic syndrome; CTCL, Cutaneous T-cell lymphoma; TCL, T-cell lymphoma; PTCL, Peripheral T-cell lymphoma; MM, Multiple Myeloma, ORR, Objective response rate.

**Table 2:** Clinical trials of epigenetic drugs alone used in solid tumors (1100 cases).

Tumor type	Year	Patient number	CR	PR	SD	PD	ORR
Epithelial ovarian or primary peritoneal carcinoma	2008	24	0	1	9	14	1
Melanoma	2014	32	0	2	16	14	2
Multiply solid tumors	2008	55	0	0	14	41	0
NSCL	2004	47	0	3	14	30	3
Soft tissue sarcoma	2013	35	0	0	7	28	0
Multiply solid tumors	2012	36	0	0	6	30	0
Refractory solid tumors	2012	39	0	1	9	29	0
Thymic epithelial tumors	2011	41	0	2	25	14	2
Multiply solid tumors	2013	92	0	1	8	83	1
Hepatocellular carcinoma	2012	42	0	10	19	13	10
Multiply solid tumors	2001	53	0	1	3	49	1
Hepatocellular carcinoma	2009	66	0	0	0	66	0
Multiply solid tumors	2006	31	0	0	1	30	0
Multiply solid tumors	2011	24	0	0	5	19	0
Pleural mesothelioma	2015	73	0	0	2	71	0
Breast cancer	2013	29	0	0	4	25	0
Multiply solid tumors	2009	18	0	0	9	9	0
Gastrointestinal cancer	2013	20	0	0	7	13	0
Thyroid Carcinoma	2009	19	0	0	9	10	0
NSCL	2009	14	0	1	7	6	1
Breast cancer	2008	14	0	4	4	6	4
Advanced solid tumors	2013	19	0	0	11	8	0
Head and neck cancer	2012	14	0	0	2	12	0
Neuroendocrine Carcinoma	2011	8	0	1	4	3	1
platinum resistant epithelial ovarian cancer and micro papillary (LMP) ovarian tumors	2010	32	0	1	10	21	1
NSCLC	2011	45	1	1	10	24	2
refractory advanced cancer	2007	26	0	0	2	12	0
advanced malignant pleural mesothelioma	2009	18	0	2	4	7	2
NSCLC	2013	8	0	0	1	33	0
Head and neck cancer	2008	9	0	0	3	6	0
Multiply solid tumors	2008	16	0	0	8	8	0
Mesothelioma	2014	5	0	0	1	4	0
Multiply solid tumors	2013	28	0	0	9	19	0
Multiply solid tumors	2011	33	0	0	10	23	0
Total		1065	1	32	263	81814	34
Response ratio (100%)			0.1	0.03	0.24	0.73	0.03

CR, complete response; PR, partial response; SD, stable disease; PD, progressive, disease; ORR (CR + PR), objective response ratio. Response identification based on. Response Evaluation Criteria In Solid Tumors (RECIST). (See references in supplementary file).

However, some studies and clinical trials applied the combined therapeutic strategy between ETD and DTD in treating solid tumors^40–45^, and many of them are still on-going as listed in Table 3. By analyzing the available literature describing such combined strategies, the beneficial effect of ETD in improving the therapeutic effect of DTD can be observed. The objective response rate (ORR) of cancer patients treated by ETD plus DTD is significantly higher than the patients treated by DTD only (Fig. 1E).

**Table 3:** On-going clinical trials of ETD plus DTD.

Identifier	First received	Cancer type	ETD	DTD	Trial phase
NCT01627041	21-Jun-12	Acute Myeloid Leukemia	Decitabine	Cytarabine,Daunorubicin Hydrochloride	II
NCT01729845	15-Nov-12	Relapsed or Refractory Acute Myeloid Leukemia or High-Risk Myelodysplastic Syndromes	Decitabine	Etoposide, and Cytarabine	I/II
NCT01829503	6-Mar-13	Acute Myeloid Leukemia	Decitabine	Cytarabine	II
NCT02452970	17-May-15	Advanced Cholangiocarcinoma	RRx-001	Gemcitabine and cisplatin	II
NCT01935947	3-Sep-13	Advanced Non-small Cell Lung Cancer	Azacitidine	Gemcitabine Hydrochloride,	II
NCT02489903	29-Jun-15	Small, Non-small Cell Lung Cancer, Ovarian Cancer and Neuroendocrine Tumors	RRx-001	Cisplatin,carboplatin, etoposide	II
NCT02159820	1-Jun-14	Ovary Cancer	Decitabine	Carboplatin	II to III
NCT02429466	14-Apr-15	Relapsed Refractory Germ Cell Tumors	SGI-110	Cisplatin	I
NCT01896856	8-Jul-13	Metastatic Colorectal Cancer	SGI-110	Irinotecan	II
NCT01386346	10-May-11	Esophageal Cancer	Azacitidine	Oxaliplatin	I

### ETD Increases the sensitivity to DTD induced apoptosis

**Figure 2:**
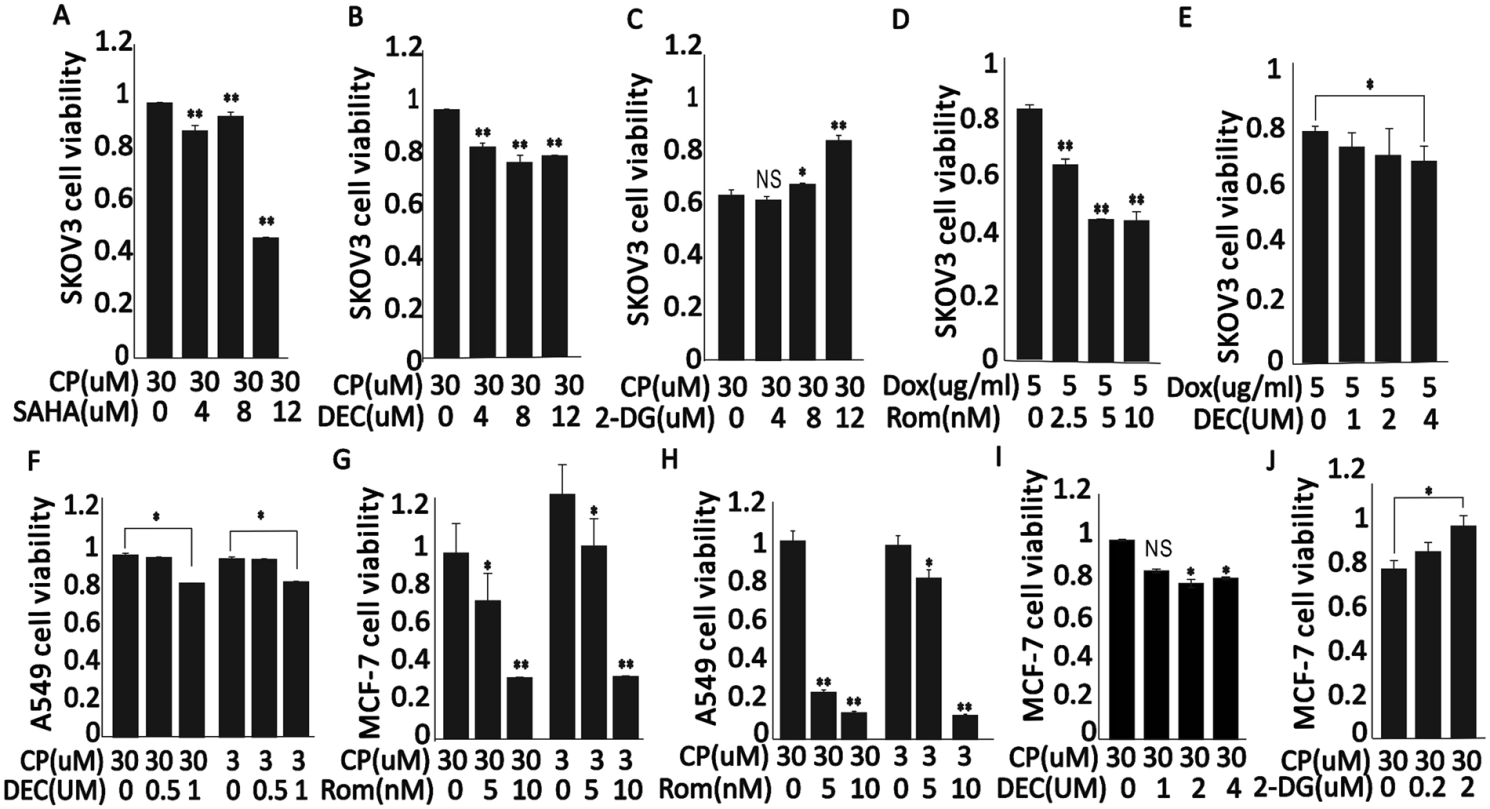
ETD Increases the sensitivity to DTD induced apoptosis. (**A**,**B** and **C**), Skov3 cell viability when cells were exposed to 30 uM CP combined with 0 uM, 4 uM, 8 uM or 12 uM SAHA for 24 h, 0 uM, 1 uM, 2 uM or 4 uM DEC for 24 h, or 0 uM, 0.2 uM, 2 uM or 20 uM 2-DG for 36 h. (**D** and **E**) Skov3 cell viability when cells were exposed to 5 ug/ml DOX combined with 0 nM, 2.5 nM, 5 nM or 10 nM Rom for 24 h or 0 uM, 1 uM, 2 uM or 4 uM DEC for 24 h. (**F**) A549 cell viability when cells were exposed to 30 uM or 3 uM CP combined with 0 uM, 0.5 uM, 1 uM DEC for 24 h. (**G**) MCF7 cell viability when cells were exposed to 30 uM or 3 uM CP combined with 0 nM, 5 nM or 10 nM Rom for 24 h. (**H**) A549 cell viability when cells were exposed to 30 uM or 3 uM CP combined with 0 nM, 5 nM or 10 nM Rom for 24 h. (**I** and **J**), MCF7 cell viability when cells were exposed to 30 uM CP combined with 0 uM, 1 uM, 2 uM or 4 uM DEC for 24 h or 0 mM, 0.2 mM, 2 mM or 20 mM 2-DG for 36 h. (* means P < 0.05, ** means P < 0.001, n ≥ 3.)

To understand the combined effects of ETD and DTD on solid tumors, several solid tumor cell lines were tested including SKOV3, MCF-7 and A549 etc. representing ovary cancer, breast cancer and lung cancer respectively. The most commonly used DTD is CP which was chosen to combine with SAHA, Rom and DEC. In addition, 2-DG was selected to potentiate the HDAC activity because 2-DG inhibits glycolysis and whole cell abundance of acetyl-CoA, the substrate for histone acetylation. As shown in Fig. 2A and B, SKOV3 cells show increased sensitivity to 30 uM CP treatment when the cells were treated together with increased SAHA or DEC in the MTT assay. 2-DG, on the other hand, increases the cell viability significantly (Fig. 2C). However, treatment of SKOV3 by DEC alone from 0 uM to 4 uM, by SAHA alone from 0 uM to 12 uM, or Rom from 0 uM to 4 uM doesn’t generate as significant impact on cell survival as when these drugs were combined with CP (Supplementary Figures 3A, 4B and C). We also tested Dox, another commonly used DTD in treating multiple types of cancer. Again, SKOV3 cells show significantly increased sensitivity to Dox when the cells were pretreated by Rom (Fig. 2D), or show minimally increased sensitivity to Dox by DEC pre-treatment (Fig. 2E). DEC also increases SKOV3 sensitivity to 3 uM CP, an extremely low concentration that hardly kills any SKOV3 cells alone^46, 47^(Fig. 2F), which is consistent to the previous studies^48, 49^. The sensitivity to CP, when cells were also treated by ETD like Rom, was also tested in MCF-7 and A549 cells. The increased sensitivity of both cells to CP can be observed Fig. 2G and H. Dead cell count also further confirmed this result (Supplementary Fig. 3D). We further demonstrated that CP, when combined with Rom or DEC, also promotes the expression of p21 and Bax, indicating the ETD and DTD drug combination inhibits cell proliferation and may increase the apoptosis pressure (Supplementary Fig. 3E). Comparably, A549, which is more resistant to CP, show dramatically increased response to CP when the cells were treated by CP and Rom or CP and DEC (Fig. 2H and I). However, Rom alone in MCF-7 cells or A549 cells didn’t generate a significant impact on cell survival (Supplementary Fig. 3F and G). Similarly, 2-DG increases the resistance of MCF-7 to CP treatment significantly (Fig. 2J). We also tested the combined effect between CP and Dox. The result shows that there is no synergistic effect between these two drugs (data not shown). Finally, MCF10A cell response to CP was evaluated with the presence of ETDs. A clear increased sensitivity was observed in MCF10A cells (Supplementary Fig. 3H and I), suggesting ETD promotes cell sensitivity to DTD is not cancer cell specific.

### ETD potentiates the DNA damage induced by DTD

**Figure 3:**
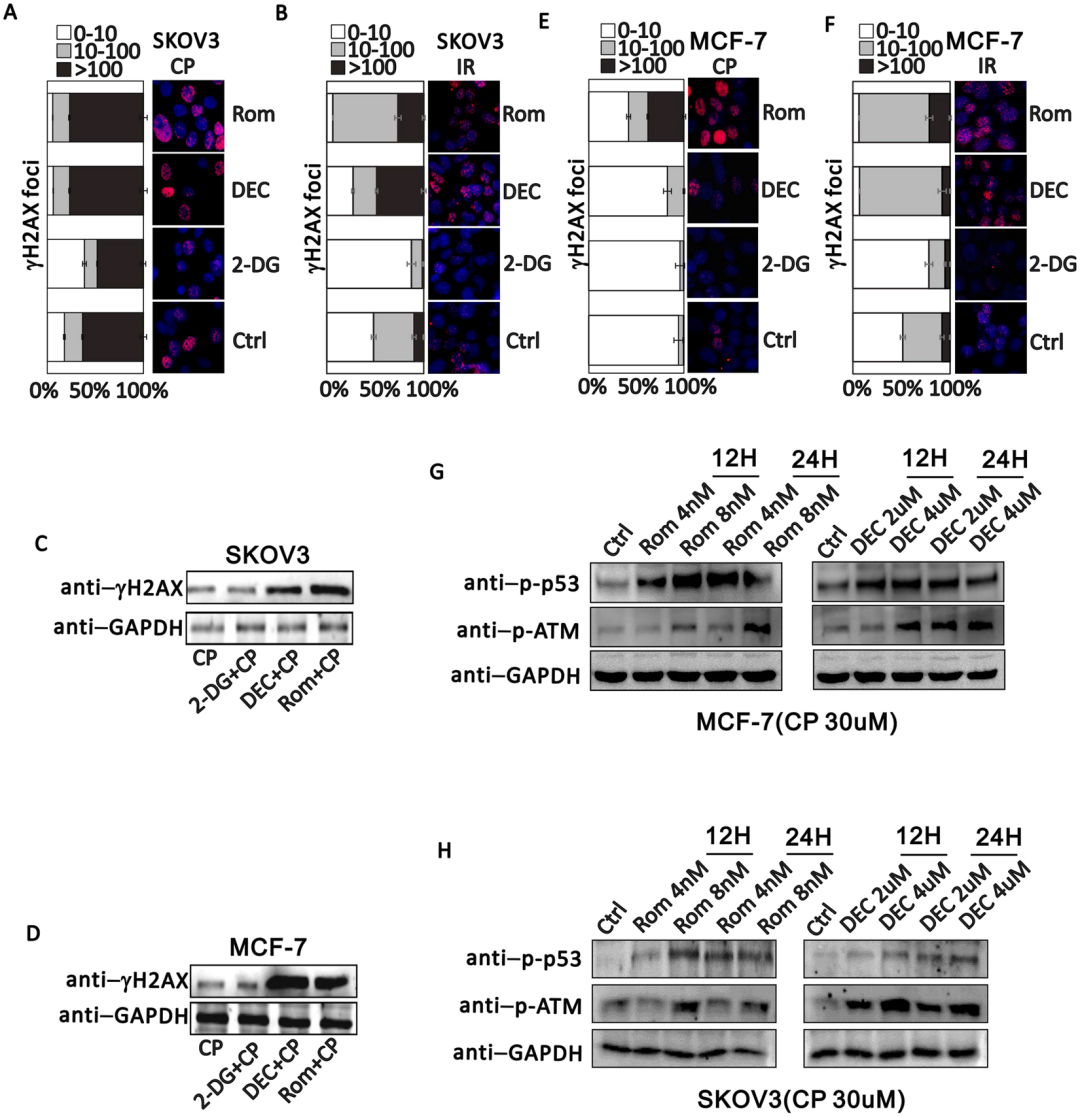
ETD increases DNA damage induced by DTD. (**A**) γH2AX immunofluorescence staining when cells were exposed to 30 uM CP alone or combining with 2.5 nM Rom, 1 uM Dec or 2 mM 2-DG in SKOV3 cells. (**B**) γH2AX immunofluorescence staining when SKOV3 cells were exposed to 10 Gy IR alone or combining with 2.5 nM Rom, 1 uM Dec or 2 mM 2-DG. (**C** and **D**) Cropped western blots show γH2AX expression level when SKOV3 cells or MCF-7 cells were treated with 30 uM CP alone or combing with 2 mM 2-DG, 1 uM Dec, or 2.5 nM Rom. Uncropped images are in Supplementary information. (**E**) γH2AX immunofluorescence staining when MCF-7 cells were exposed to 30 uM CP alone or combining with 2.5 nM Rom, 1 uM Dec or 2 mM 2-DG. (**F**) γH2AX immunofluorescence staining when MCF-7 cells exposed to 10 Gy irradiation alone or combining with 2.5 nM Rom, 1 uM Dec or 2 mM 2-DG. (**G** and **H**) Western blotting detection of phosphorylated p53 (p-p53) and phosphorylated ATM upon treatment of MCF-7 (**G**) and SKOV3 (**H**) by Rom (left) or DEC (right). GAPDH serves as an endogenous control. 2 time scale (12 H, 12 hours treatment; 24 H, 24 hours treatment) and 2 dosage (Rom, 4 nM and 8 nM; DEC, 2 uM and 4 uM) were applied.

Lethal DNA damage is the main reason of cell death induced by CP and Dox^27, 28^. CP induces DNA breaks by creating inter-strand adducts^27^. Dox also creates DNA damage through being incorporated into genome DNA^27^. Dox also creates DNA damage through incorporating into genome DNA^28^. Comparing to CP or IR treated SKOV3 cells, both DEC and Rom potentiates the CP or IR-induced DNA damage represented by phosphorylated γH2AX foci detected by immunocytochemistry (Fig. 3A and B). On the contrary, 2-DG decreases the CP or IR-induced γH2AX foci (Fig. 3A and B). Western blot of total phosphorylated γH2AX also indicates that ETD increases the DNA damage generated by CP in both SKOV3 and MCF-7 cells (Fig. 3C and D), suggesting the CP-induced DNA damage is under influence of ETDs without tumor specificity. Again, phosphorylated γH2AX show mild decrease in 2-DG treated samples which represent mild protection to the cells exposed to CP (Fig. 3C and D). Similarly, DEC and Rom also increases the DNA damage generated by CP or IR in MCF-7 cells significantly and 2-DG decreases the sensitivity to CP or IR in MCF-7 cells (Fig. 3E and F). To further demonstrate that ETD and DTD drug combination increases the DNA damage, the phosphorylated p53 and phosphorylated ATM were detected to monitor if there is activation of DNA damage repair pathways. As shown in Fig. 3G and H, both MCF-7 and SKOV3 cells show increased phosphorylated p53 and phosphorylated ATM upon treatment by CP and Rom or CP and DEC. However, there is no dramatic increase when the treatment was extended from 12 hours to 24 hours, suggesting the DNA damage response is within a short period of time. These results are consistent to the previous report that SAHA treatment increases the nuclease sensitivity and intercalating agent sensitivity^50^.

### ETD increases the DTD accessibility

**Figure 4:**
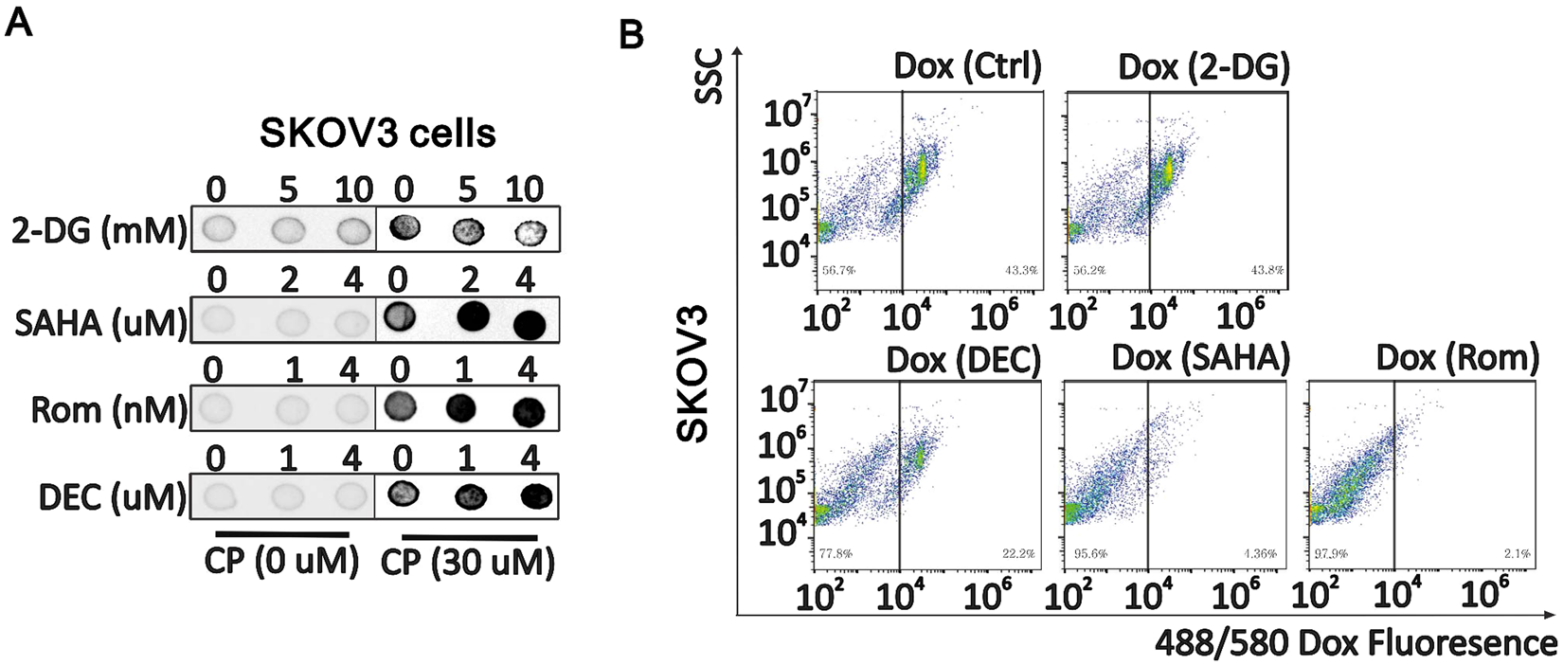
ETD increases DTD accessibilities in chromosome. (**A**) Dot blot analysis of CP-DNA adduct when SKOV3 cells were treated with 2-DG, SAHA, Rom or DEC with indicated concentration for 6 h, or SKOV3 cells were treated with a combination of CP 30 uM 4 h and 2-DG, SAHA, Rom or Dec with indicated concentration for another 6 h. (**B**) Flow cytometer detection of DOX autofluorescence in nuclei from cells treated with Dox alone or combined with 2-DG, DEC, SAHA or Rom. X axis indicates the 488/580 Dox fluorescence value, Y axis indicates SSC.

Since DNA damage generated by CP and Dox relies on the attachment of CP and Dox to DNA directly, we attempted to observe if ETD increases the CP and Dox integration into DNA. The antibody recognizing specifically the CP-DNA adducts was used to detect the CP molecules which covalently bound to DNA, but not the free CP. By extracting genomic DNA from CP treated cells and detecting the CP-DNA adducts via dot-blotting, the retention of CP is revealed (Supplementary Fig. 4A). As shown in Fig. 4A, increasing the SAHA, Rom and DEC concentration in treating SKOV cells results in the increased CP-DNA adduct abundance and 2-DG, on the contrary, reduces the CP-DNA adducts abundance. Dox is an auto-fluorescent dye with an excitation/emission wavelength at 488/580 nm. However, the fluorescence signal of Dox extinguishes once forming stable bound with DNA^51^. By isolating nucleus and measuring their fluoresce signal at 580 nm through flow cytometry assay, the amount of Dox that forms stable covalent bound with DNA in each individual nucleus can be estimated. As shown in Fig. 4B, the vertical straight line in each panel indicates the Dox fluorescence signal to be high (right) or low (left). Given the total nucleuses that were counted, the treatment dosage of Dox are all the same across all the panels, and the total nuclear absorption of Dox is not influenced by the drug treatment (Supplementary Fig. 4B), the increase of nucleus population with low Dox signal in DEC, SAHA and Rom pretreatment groups indicates that more Dox forms stable bound to DNA. 2-DG pretreatment, however, increases the population of nucleuses with high Dox signal. These results suggest ETDs increase the retention of both CP and Dox to the chromatin which is the direct reason of increased DNA damage.

### ETD decompact the chromatin globally

**Figure 5:**
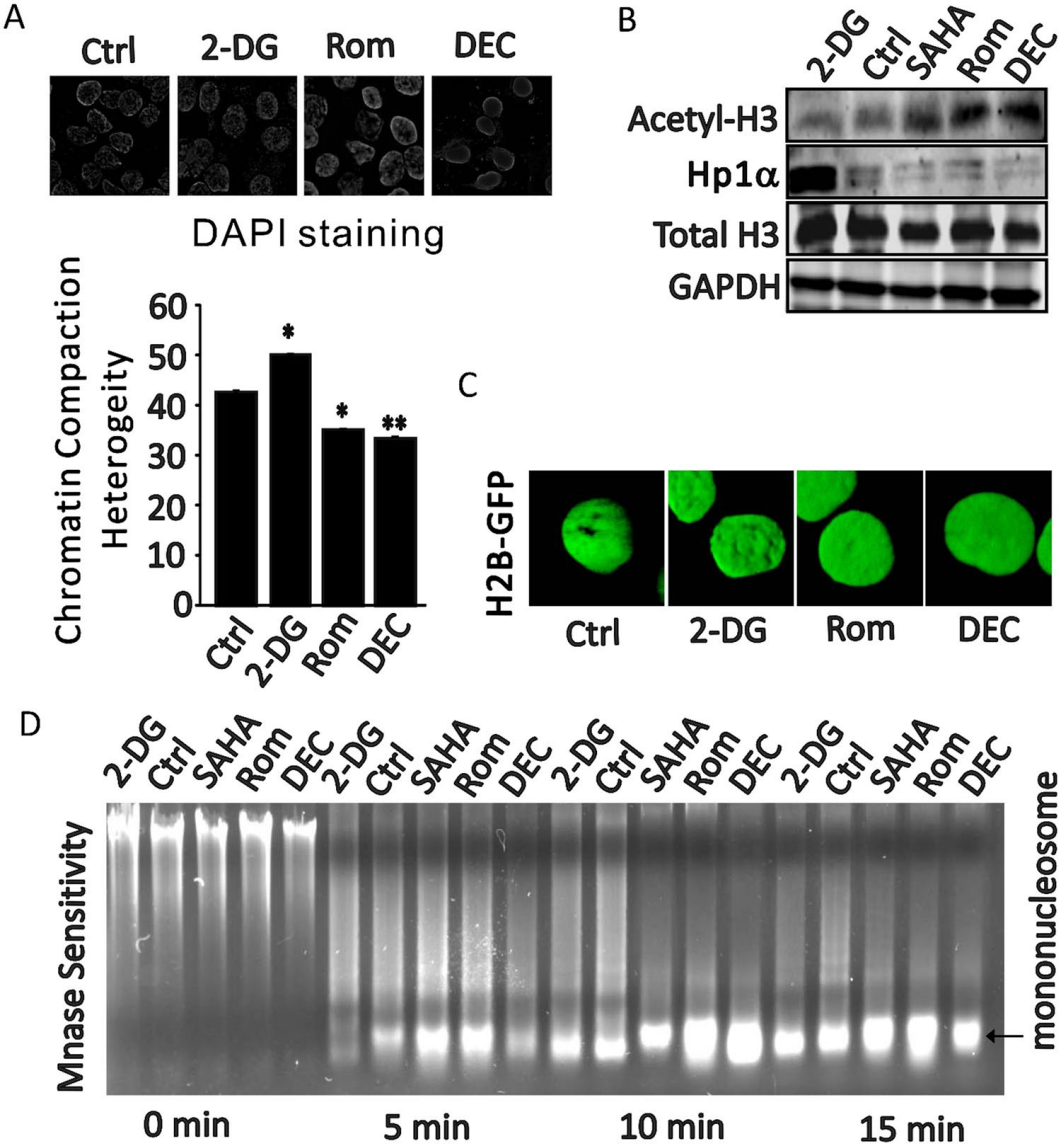
Epigenetic drugs affect global chromosome compaction. (**A**) Nuclear chromatin compaction heterogeneity by DAPI staining (top) in SKOV3 cells treated with ethanol, 2 mM 2-DG, 1 uM DEC or 2.5 nm Rom for 12 h. The bottom chart is the quantitation of the chromatin compaction heterogeneity. (**B**) Cropped western blots show acetyl-H3 expression and chromatin bound HP1α level when SKOV3 cells were treated with ethanol, 2 mM 2-DG, 4 uM SAHA, 2.5 nM Rom or 1 uM DEC. Uncropped western blots are in Supplementary information. (**C**) SKOV3 cells were stably transfected with GFP-H2B plasmid, then cells were treated with ethanol, 10 uM 2-DG, 1 uM DEC or 2.5 nM Rom for 12 h. 3D-reconstruction shows the H2B distributions in nucleus representing the chromatin 3D conformation. (**D**) MNase assay shows mononucleosome bound DNA quantity when cells treated with ethanol, 2-DG, SAHA, Rom and DEC for 0 min, 5 min, 10 min, and 15 min.

Chromatin compaction, either locally or globally, is the main target of ETDs^52^. We hypothesized that ETD may potentiate the effectiveness of DTD through loosening chromatin globally which is required for DTD incorporation and DNA damage as seen in Fig. 4A and B. DAPI, a commonly used dye in DNA staining, was chosen to demonstrate the compactness of nuclear DNA using SKOV3 cell^53^. By limiting the dosage and time of DAPI staining, the florescence emission shows patchy distribution within the nucleus in control cells, indicating co-existence of highly compacted chromatin and less compacted chromatin (Fig. 5A). 2-DG increases the abundance of heavily stained chromatin dramatically, whilst DEC and Rom almost erase all the heavily stained chromatin (Fig. 5B), suggesting these drugs are capable of modulating the chromatin compaction globally^54, 55^, which is consistent with the effect generated by TSA treatment^56^.

To further validate the global chromatin compaction status influenced by ETD, we extracted histones from ETD treated nucleuses^57^. Acetylated histone H3, a marker of loose chromatin, show consistent upregulation by SAHA, Rom and DEC but not by 2-DG (Fig. 5B Top). Another independent protein to indicate the chromatin compaction is HP1α which is a heterochromatin binding protein and its abundance correlates with the global change of heterochromatin and was used for evaluating global chromatin compaction^58, 59^. As shown in Fig. 5B (bottom), chromatin-bound HP1α significantly decreased by SAHA, Rom and DEC but dramatically increased by 2-DG. These data suggest that ETDs treatment alters both the active chromatin and repressive chromatin simultaneously and globally.

In addition, the SKOV3 cell stably expressing fusion H2B-GFP was used to evaluate the effect of ETDs on nuclear chromatin compaction^58, 60^. As shown in Fig. 5C, H2B-GFP signal in nuclei of Rom and DEC treated cells are more flatten and even, comparably, 2-DG treatment seems increasing the local compaction of chromatin in some area.

Finally, to directly demonstrate the change of chromatin compaction at DNA level, MNase sensitivity assay was performed^61^. As shown in Fig. 5D and Supplementary Fig. 5, SAHA, Rom and DEC consistently increase the chromatin sensitivity to MNase and the mono-nucleosome bound DNA but not 2-DG, suggesting ETD is able to decompact the chromatin globally.

### ETD improves the therapeutic effect of DTD

**Figure 6:**
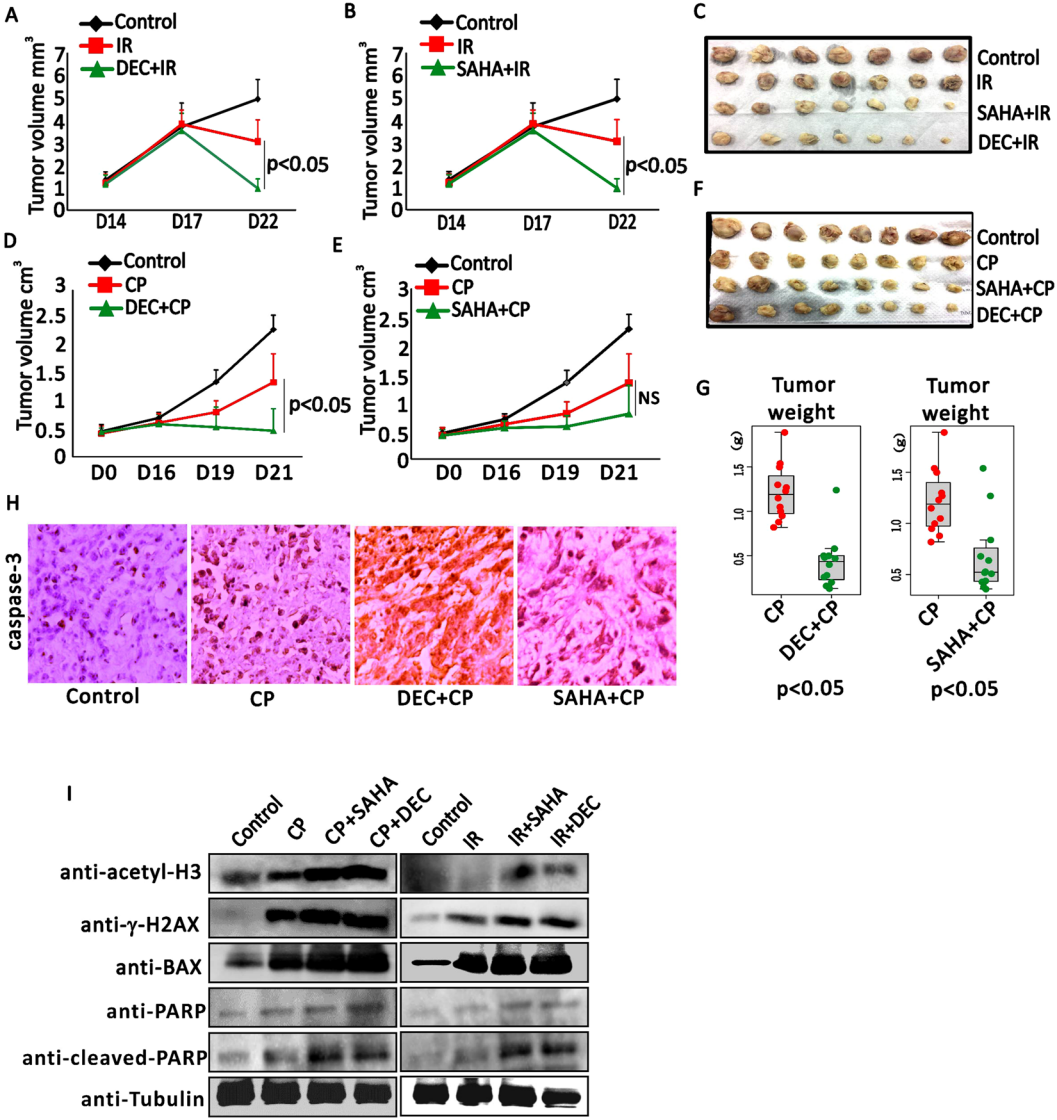
ETD promotes DTD therapeutic effect in engrafted tumors. (**A**) Growth curves of xenograft tumors in mice administrated with solvent (control), IR and SAHA combined with IR, (N = 12). (**B**) Growth curves of xenograft tumors in mice treated with solvent (control), IR and DEC combined with IR (N = 12). (**C**) Tumors are shown after being dissociated from mice with indicated conditions. (**D**) Tumor growth curves of xenograft tumors in mice treated with solvent (control), CP and DEC combined with CP, N = 12. (**E**) Growth curves of xenograft tumors in mice treated with solvent (control), CP and SAHA combined with CP (N = 12). (**F**) Tumors are shown after being dissociated from mice with indicated conditions. (**G**) The box plots show the mean tumor weights ± SEM (N ≥ 10) with indicated treatments including CP, CP combined with DEC or SAHA. (**H**) Representative caspase-3 histochemical staining of xenograft tumors treated with solvent (control), CP, SAHA combined with CP and DEC combined with CP. (**I**) Western blotting detection of acetylated H3, gH2AX, BAX, PARP and cleaved-PARP upon the cells are treated by CP/IR, or CP/IR plus SAHA/DEC, using tubulin as endogenous control, in mouse engrafted tumors.

To further validate the hypothesis that ETD increases the ability of DTD in killing cancer cells, the breast tumor model was established using SCID mice. 4T1 is a malignant breast cancer cell line originated from mice^62^ and was used to establish the tumor models. Both DEC and SAHA significantly retard the tumor growth along the treatment by IR in these engrafted breast tumors (Fig. 6A,B and C). For the tumors exposed to CP treatment, both DEC and SAHA improves the CP effect in shrinking the tumor significantly (Fig. 6D,E and F). Statistics of the tumor weight using the box plot indicates that DEC and SAHA significantly improve the tumor sensitivity to CP (Fig. 6G). Importantly, the western blotting of acetylated H3 increased in tumors treated by CP plus SAHA or DEC, and IR plus SAHA or DEC dramatically, along with the increased DNA damage response signals such as γ-H2AX and cleaved PARP. Also, the apoptosis signal is dramatically increased in these tumors treated with drug combination (Fig. 6I). Like CP, Dox also show a significant synergistic effect when either DEC or SAHA were used to treat the tumor bearing mice (Supplementary Fig. 6). Together, these data strongly indicate that ETD potentiates the therapeutic effect of DTD via increasing the DNA damage generated by DTD.

## Discussion

Application of ETD in tumor therapy is supported by several important observations including re-initiating the expression of tumor suppressor genes^10, 23^, improving the radio-sensitivity of cancer cells^31, 43, 63^, blocking the DNA repair activity^64^, and repressing expression of DNA repair genes etc.^9^. However, the investigation on the clinical effectiveness of ETD suggests only very limited subtypes of cancer can be treated by this category of drugs effectively and these cancer mainly is hematopoietic cancers, suggesting that ETD treatment only minimally influences the cell survival^24^. Theoretically, ETD mainly targets the factors that regulate the chromatin status and their effect can be much mild by comparing to DTD. However, up-regulation of HDACs and DNMT genes is very common among the human cancers. So ETD becomes the attractive drug that can be broadly used to treat different types of cancer and many clinical trials are still on-going^23^.

Recovery of tumor suppressor expression was particularly addressed in the cancer subtypes indicated by the ETD drug licenses. However, quite many studies point out the discrepancy that ETD may not only turn on the tumor suppressors but also turn on other genes such as the pro-metastasis genes^65^. Simultaneously, DNA repair genes are repressed by ETD treatment in multiple types of cancer cells^7, 9, 66, 67^, suggesting tumor suppressor genes are not the only target of ETD. These combined effects generated by ETD treatment also raises confusion in understanding the mechanism through which ETD treatment increases the therapeutic effect of DTD^17, 63, 67–70^.

In our current study, we propose that ETD globally influences chromatin structure. Although previous studies have pointed out the possibility that global change of chromatin organization is responsible for the increased therapeutic effect generated by ETD and DTD combination, the evidence to directly demonstrate that ETD potentiates DNA damage induced by DTD is absent. For example, TSA has been shown to induce the global chromatin decompaction and leads to a more homogenous distribution^56^. Importantly, such chromatin decompaction has no gene locus specificity because image analysis indicates that the chromatin reorganization occurs to chromatin domains crossing several megabases of DNA, which equivalent to 200 nm to 1 um of change under microscope^56^. In another independent study, VPA treatment evacuates several important factors that are critical to maintaining the higher order chromatin structure such as the members of cohesion and condensins including SMC proteins, SMC-associated proteins and some heterochromatin proteins^50^.

Falk *et al*., for the first time, correlates the chromatin condensation to irradiation sensitivity directly^31^. This study suggested highly compacted chromatin is more resistant to DNA damage induced by irradiation. This study was followed by others which recap the similar results using different ETDs such as SAHA, 5-aza-CdR, DEC, and TSA etc.^17, 43, 68^. In fact, several recent studies validated that DTD is influenced by ETD for their DNA damage induction and cell death induction. For example, SAHA increases the apoptosis of Rhabdomyosarcoma cell death when treated together with Dox^70^. A recent study also systematically investigated the synergistic effect of CP and HDAC inhibitors using several cell models and the result is also very promising^71^. Interestingly, another independent study observed the enhanced sensitivity of DNA to nucleases and increased interaction of DNA with intercalating agents simultaneously after the cells were treated by ETDs^50^. All these observations strongly support that ETDs are able to potentiate the therapeutic effect of DTD by loosening the chromatin and increasing the DNA damage sensitivity.

The other benefit of ETD and DTD combined treatment of tumor is to reduce the drug resistance as observed in our data (Fig. 2). Traditionally, the main reason of drug resistance is the appearance of DTD resistant cancer cell population after a period of tumor therapy. The mechanism of drug resistance is complicated, but the dosage limitation of DTD is critical since the patient tolerance have to be counted. The dosage lower than the threshold to kill all the tumor cells will leave some cells alive which eventually grow as drug resistance colonies. ETD treatment, however, will program the cells to a more vulnerable status that can be killed by even low dosage of DTD. In other words, the threshold to kill the majority of tumor cells is lowered by ETD co-treatment. Therefore, an extra benefit to the patients will be reduced DTD toxicity and the associated side effect because of the lower DTD dosage in addition to the higher efficiency in killing tumor cells.

## Materials and Methods

### Gene enrichment analysis

The total tumor suppressors and oncogenes were obtained by searching Uniprot by the following keywords: Tumor suppressor keywords are “Tumor suppressor [KW-0043]” AND organism:” Homo sapiens (Human) [9606]”; oncogene keywords are “Oncogene [KW-0553]” AND organism:” Homo sapiens (Human) [9606]”+keyword: “Proto-oncogene [KW-0656]” AND organism: “Homo sapiens (Human) [9606]”. Then we mapped to gene official name using ID mapping tool on the David gene ID conversion tool. Totally 236 oncogenes and 169 tumor suppressors are obtained. The consensus upregulated oncogenes and tumor suppressor genes were obtained by checking their expression cross all 18 primary AML samples (GSE40442) treated with DEC. Chi-square test was used to test the significance.

### Meta-analysis

We searched clinical trials of epigenetic targeting drugs (ETD) published before the date of Dec, 31, 2016 and selected the randomized and double blind trials and got 4 candidate research articles. We integrated all these trials and collected 119 cases in total. We calculated the pooled unadjusted *odds ratio* with 95% CIs for each study using a random-effects model. Analysis was performed with Revman software.

### Cell culture and chemicals

The human cancer cell lines MCF7, SKOV3 and A549 were all from ATCC. All cells were cultured in Dulbecco’s Modified Eagle’s medium (Gibco) supplemented with 10% fetal bovine serum (Gibco) and 1% penicillin /streptomycin (Gibco) in a humid atmosphere containing 5% CO2 at 37 °C. Cell synchronization is through the serum starvation for 24 h. 2-DG(D8375), SAHA(SML0061), Romidepsin(SML1175) and Decitabine (189825) were all ordered from Sigma; Cisplatin (S1552) was ordered from Beyotime (Shanghai, China). Doxorubicin solution was ordered from Hisun-pfizer Company.

### MNase Assay

Briefly, the MNase assay was performed according to liu *et al*.^31^. Cells pellets were lysed in a hypotonic buffer (10 mM Tris-HCl (PH 7.4), 10 mM KCl, 15 Mm MgCl2) on ice for 10 min. then nuclei were centrifuged and re-suspended in Micrococcus nuclease (M0247,NEB) (Mnase) digestion buffer (0.32 M sucrose, 50 mM Tris-HCl (PH 7.5), 4 mM MgCl2, 1 Mm CaCl2, 0.1 mM pheylmethylsulfony fluoride(PMSF) supplemented with 1*BSA and 10 U MNase for each sample at 37 °C. The MNase reaction was terminated by addition of 10 mM EDTA followed by centrifugation. The nuclear pellets were then re-suspended in MNase digestion buffer supplemented with RNase and incubated at 37 °C for 30 min. DNA fraction was purified as regular method, and then run 1.2% agarose gel, the gel images were captured with image system (ChemiDoc™ Touch, Biorad) and quantities analyzed with Image J.

### Cisplatin dot blot assay

Skov3 cells were pretreated with different concentration of Cisplatin 0 uM, 3 uM, 5 uM, 10 uM or 30 uM, or indicated concentration of DMSO, 2-DG, SAHA, Rom or Dec for 6 h, then all cells were treated with 30 uM cisplatin. Following extraction of genomic DNA, the samples were sonicated for 5 min with 30 s on/30 s off. Then the concentration of each sample was measure with Nanodrop 2000 (Thermo Scientific). Samples in the same comparative group were diluted to same concentration. Drop equal volume (2 ul or 3 ul) samples to a nitrocellulose filter and bake the filter on 65 °C for 30 min following with UV crosslink (120000 J, 3 min).

### Animal assay

Experimental setup for irradiation therapy part: 1*10^6^ 4T1 breast cancer cells were inoculated into subcutaneous of SCID mice hind limb on Day 1. Mice were randomly divided into three groups on Day 14. For each group, N = 12 tumors. The mice in ETDs combined with IR groups received 0.4 mg/kg Dec or 30 mg/kg SAHA intravenous injection via tail vein on Day 15, 16, 17, at the same time control and IR group receive solvent injection. All mice received 10 Gy irradiation on D18. Experimental terminal is D22. Tumor volume was measured and calculated by the formula V = (length × Width^2) × π/6. Tumors were surgically removed from mice on Day 22.

For cisplatin therapy part: 1*10^6^ 4T1 breast cancer cells were inoculated into subcutaneous of SCID mice hind limb on Day 1. Mice were randomly divided into three groups on Day 14. For each group, N = 12 tumors. The mice in ETDs combined with cisplatin groups received 0.4 mg/kg Dec or 30 mg/kg SAHA intravenous injection via tail vein on Day 14, 15, 17, 18, at the same time control and cisplatin group received solvent injection. All mice received 5 mg/kg cisplatin on D16 and D18. Experimental terminal is D21. Tumor volume was measured and calculated by the formula V = (length × Width^2) × π/6. Tumors were surgically removed from mice on Day 22.

For Doxorubicin therapy, the Doxorubicin dosage was 10 mg/kg intravenous injection via tail vein. Other time and process was similar with cisplatin therapy design.

All the animal related experimental procedures were approved by Animal Ethics Committee of University of Macau (AECUM) and the related experiments were conducted in accordance with the guideline of AECUM.

### Immunohistochemistry staining assay

Paraffin-embedded specimens were de-paraffinized in xylene, subjected to heat-mediated antigen-retrieval in antigen unmasking solution(H-3300, Vector Laboratories), permeabilized in 0.2% Triton X-100 (Sigma), blocked in 5% goat sera. Apoptosis was detected using a mouse monoclonal anti-caspase 3 antibody (Beyotime, China) (1:50) and an HRP-conjugated donkey anti-rabbit secondary (1:250, sigma), detected using DAB reagent (JK-4100, Vector Laboratories). Then stained with HE Dye (Beyotime, China) following with dehydration and mounting. Images were acquired using x-cite 120 (Olympus) microscope.

MTT assay, Typan blue staining assay and Western blot assay are all in supplementary.

### Experimental Statistical Analyses

Results for continuous variables are presented as means ± standard deviation (SD) unless stated otherwise. All data are evaluated by 2-tailed student t-test and P < 0.05 was considered statistically significant.

## Electronic supplementary material

Supplementary Information
