# Supplementary material for: Epigenetic targeting drugs potentiate chemotherapeutic effects in solid tumor therapy
Source: Sci Rep. 2017 Jun 22;7:4035. doi: 10.1038/s41598-017-04406-0 (PMC5481380; doi:10.1038/s41598-017-04406-0)
Supplement: Supplementary file 1 — Supplementary Information [file 41598_2017_4406_MOESM1_ESM.doc]

**Epigenetic targeting drugs potentiate chemotherapeutic effects in solid tumor therapy**

Jingjing Li^1^, Dapeng Hao^1^, Li Wang^1,2^, Haitao Wang^1^, Yuan Wang^1^, Zhiqiang Zhao^1^, Peipei Li^1^, Chuxia Deng^1^, Li-jun Di^1＊^,

**1. Cancer Center, Faculty of Health Sciences, University of Macau**

**2. Metabolomics Core, Faculty of Health Sciences, University of Macau**

Correspondence should be addressed to Prof. Li-jun Di ([lijundi@umac.mo](mailto:lijundi@umac.mo)), Cancer Center, Faculty of Health Sciences, University of Macau, E12, Avenida da Universidade, Taipa, Macau, SAR of People’s Republic of China

**Supplementary Material and Methods**

**Gene enrichment analysis**

The total tumor suppressors and oncogenes were obtained by searching Uniprot by the following keywords: Tumor suppressor keywords are "Tumor suppressor [KW-0043]" AND organism:" Homo sapiens (Human) [9606]"; oncogene keywords are "Oncogene [KW-0553]" AND organism:" Homo sapiens (Human) [9606]" + keyword: "Proto-oncogene [KW-0656]" AND organism: "Homo sapiens (Human) [9606]". Then we mapped to gene official name using ID mapping tool on the David gene ID conversion tool. Totally 236 oncogenes and 169 tumor suppressors are obtained. The consensus upregulated oncogenes and tumor suppressor genes were obtained by checking their expression cross all 18 primary AML samples (GSE40442) treated with DEC. Chi-square test was used to test the significance.

**DAPI Staining Assay**

Cells were seeded in 8-well chamber (Nunc, Lab Tek) and incubated at 37°C for 24h. Then cells are exposed to indicate chemicals treatment before washed by cold PBS and fixed with pre-chilled 4%PFA for 15 min at 4°C. After blocking with 1% bovine serum albumin in PBS-Triton 0.1%, cells were mounted in VECTASHIELD® Mounting Medium with DAPI (Vector Laboratories, Burlingame, CA) and analyzed using Zeiss confocal microscope (Zeiss LSM 710).

**MTT Assay**

Cells were seeded in 96-well flat-bottom culture plates. After incubation with indicated treatment, the medium was aspirated and cells were treated with MTT (M5655, Sigma) containing medium for 4h. Then, the unreduced MTT solution was discarded, and DMSO (0.1 ml) was added into each well of the reduced MTT solution to dissolve the purple formazan precipitate , then OD values were detected with 550 nm filter of Victor X5 (Perkin Elmer, US).

**Immunofluorescence staining**

Cells were seeded in 8-well chamber and incubated at 37°C for 24h. Then cells are treated by indicated chemicals before washed by cold PBS and fixed with pre-chilled 4%PFA for 15 min at 4°C. After blocking with 1% bovine serum albumin in PBS-Triton 0.1%, cells were incubated with primary antibodies: γH2AX (Cat. 05636, Millipore), cisplatin (Cat.103261, Abcam) overnight at 4°C. Then cells were washed three times with PBS-Triton 0.1% and incubated with Alexa-Fluor-conjugated secondary antibodies against mouse or rat (Invitrogen) at room temperature for 1 hour in the dark. Cells were mounted in VECTASHIELD® Mounting Medium with DAPI and analyzed using Zeiss confocal microscope (LSM710).

**Trypan blue staining assay**

Trypan blue exclusion assay was performed by mixing 200 μL of cell suspension with an equal amount of 0.3% Trypan blue solution (Sigma, St Louis, MO) in PBS. After 5 min incubation at room temperature, the number of cells including Trypan blue (stained) was counted using a hemocytometer to estimate dead cell percentage. Each measurement was repeated three times independently.

**Western Blot Analysis**

Briefly, whole-cell proteins were generated by RIPA buffer (Beyotime). Proteins were extracted (O'Hagan, Wang et al. 2011) and separated by [polyacrylamide gel electrophoresis](https://en.wikipedia.org/wiki/Polyacrylamide_gel_electrophoresis) with SDS. Then the proteins were probed with primary antibodies, γH2AX (05636, Millipore), GAPDH (sc-48166, Santa Cruz), H3 (sc10809, Santa Cruz), P21 (sc-756, Santa Cruz), tublin (sc-5286,Santa Cruz), Phospho-p53 (Ser15) (9286,CST), Phospho-ATM(Ser1981) (AA866,Beyotime), Bax (Ab026,Beyotime), caspase (AC030,Beyotime), parp (5625,CST ),after being transferred to the PVDF membrane. Then the membrane was incubated with Dylight®-conjugated secondary antibody (Thermo), then visualized with Odyssey CLX infrared imaging system (Li-Cor Biosciences) or HRP-conjugated secondary antibody (Sigma), and visualized with ECL Prime Western Blotting Detection Reagent (GE) and pictures were taken with image system (ChemiDoc™ Touch, Biorad).

**Supplementary Figure1**

**Figure S1A. Gene enrichment analysis of tumor suppressor and oncogene in GSE40442.** At top, green indicates the genes with up-regulated expression after treatment; red indicates the genes with down-regulated expression after treatment. Middle, each blue bar stands for a tumor suppressor gene and each pink bar stands for an oncogene. Bottom part shows the gene enrichment.

**Supplementary Figure2**

Figure S2 Pan-cancer DNMT1 and HDAC1 expression (cBioportal)

Each dot represents a tumor sample and its Y-axis position represents the normalized expression of DNMT1 (top) or HDAC1 (bottom). Box plot is to indicate the difference between different types of cancers for DNMT1 or HDAC1 expression.

**Supplementary Figure3**


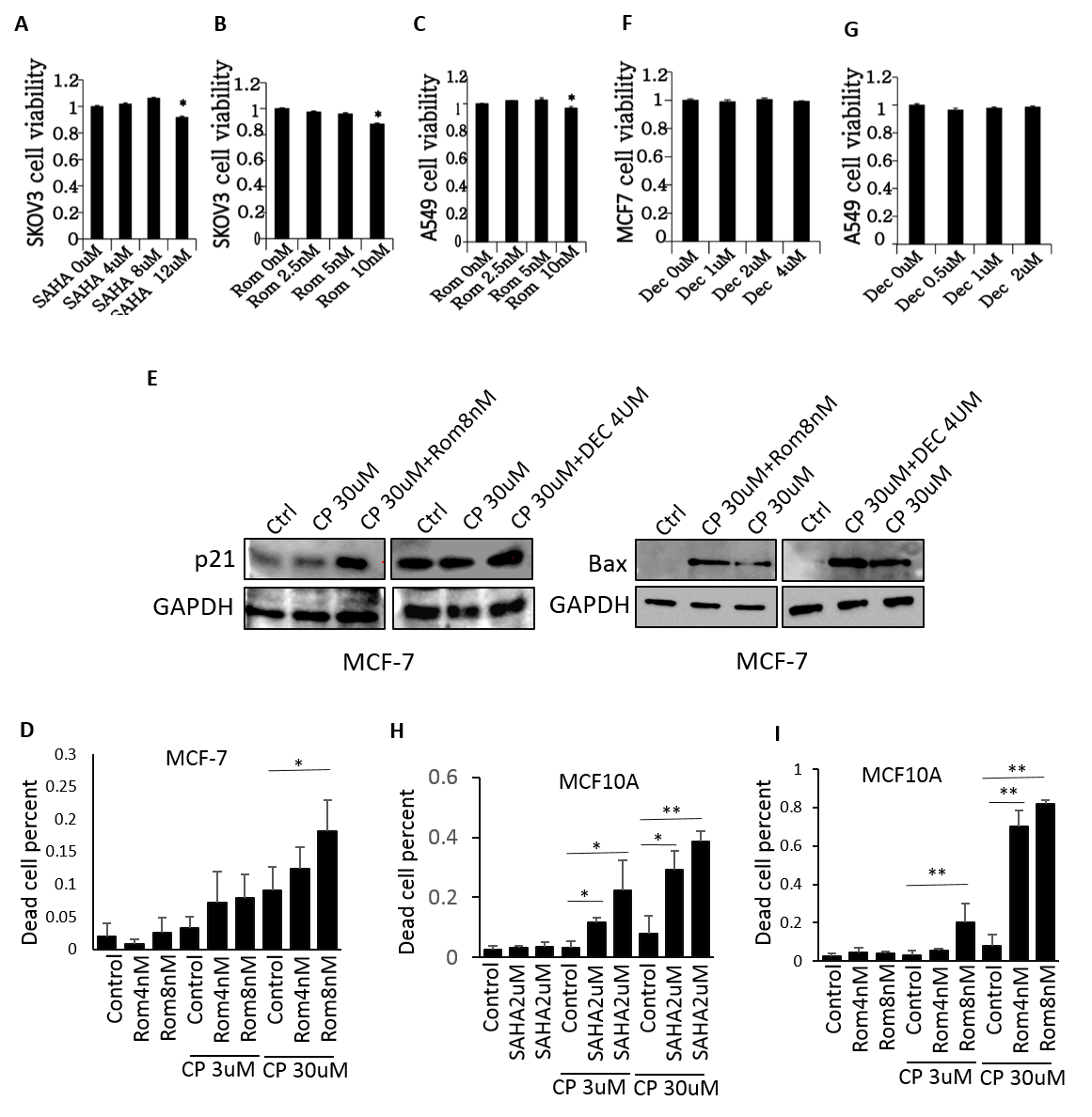


**Figure S3 Cell viabilities when cells expose to epigenetic drugs alone.**

A. Skov3 cell viability when cell exposed to SAHA 0uM, 4uM, 8uM and 12uM. **B.** Skov3 cell viability when cell exposed to Rom 0nM, 2.5nM, 5nM and 10nM. **C.**  A549cell viability when cell exposed to Rom 0nM, 2.5nM, 5nM and 10nM. **D.** Trypan blue staining shows the dead cell percentage when MCF7 cells exposed to control, Rom 4nM, Rom 8nM alone or combined with CP 3uM and 30uM respectively.  **E.** Cropped western blot shows BAX and P21 expression when MCF7 cells exposed to control, 30uM CP and 30uM CP combined with Dec or Rom for 24h respectively. (N=8) **F.** MCF7 cell viability when cell exposed to Dec 0uM, 1uM, 2uM and 4uM. **G.** A549 cell viability when cell exposed to Dec 0uM, 0.5uM, 1uM and 2uM. **H.** Trypan blue staining shows the dead cell percentage when MCF10A cells exposed to control, SAHA 2uM, SAHA 4uM alone or combined with CP 3uM and 30uM respectively. (N=4) **I.** Trypan blue staining shows the dead cell percentage when MCF10A cells exposed to control, Rom 4nM, Rom 8nM alone or combined with CP 3uM and 30uM respectively.(N=4)

**Supplementary Figure4**

**Figure S4 Retention of CP to genomic DNA**

**A.** Dot blotting is applied to detect the genome bound CP after DNA was extracted from CP treated cells. The working dosage of CP is labeled along the blot. **B.** Dox auto-fluorescence detection When cells are exposed to Dox 1ug/ml or 5ug/ml alone or combined with 2-DG, Dec, Rom and SAHA. Y axis reflects the relative florescence value. ns, none significance when compared to Ctrl.

**Supplementary Figure5**

**Figure S5. Quantitation of Mnase assay**

Top, 2-DG treatment for 0, 5, 10, and 15 mins by comparing to the control group. Bottom, SAHA, Rom and DEC treatment for 0-, 5, 10, 15 mins by comparing to the control group.

**Supplementary Figure6**

**Figure S6 Mean tumor volumes ± s.e.m. of mice with tumor xenografts (N≥10) administrated with Dox, Dox combined with Dec or SAHA.**

**Supplementary Figure 7**


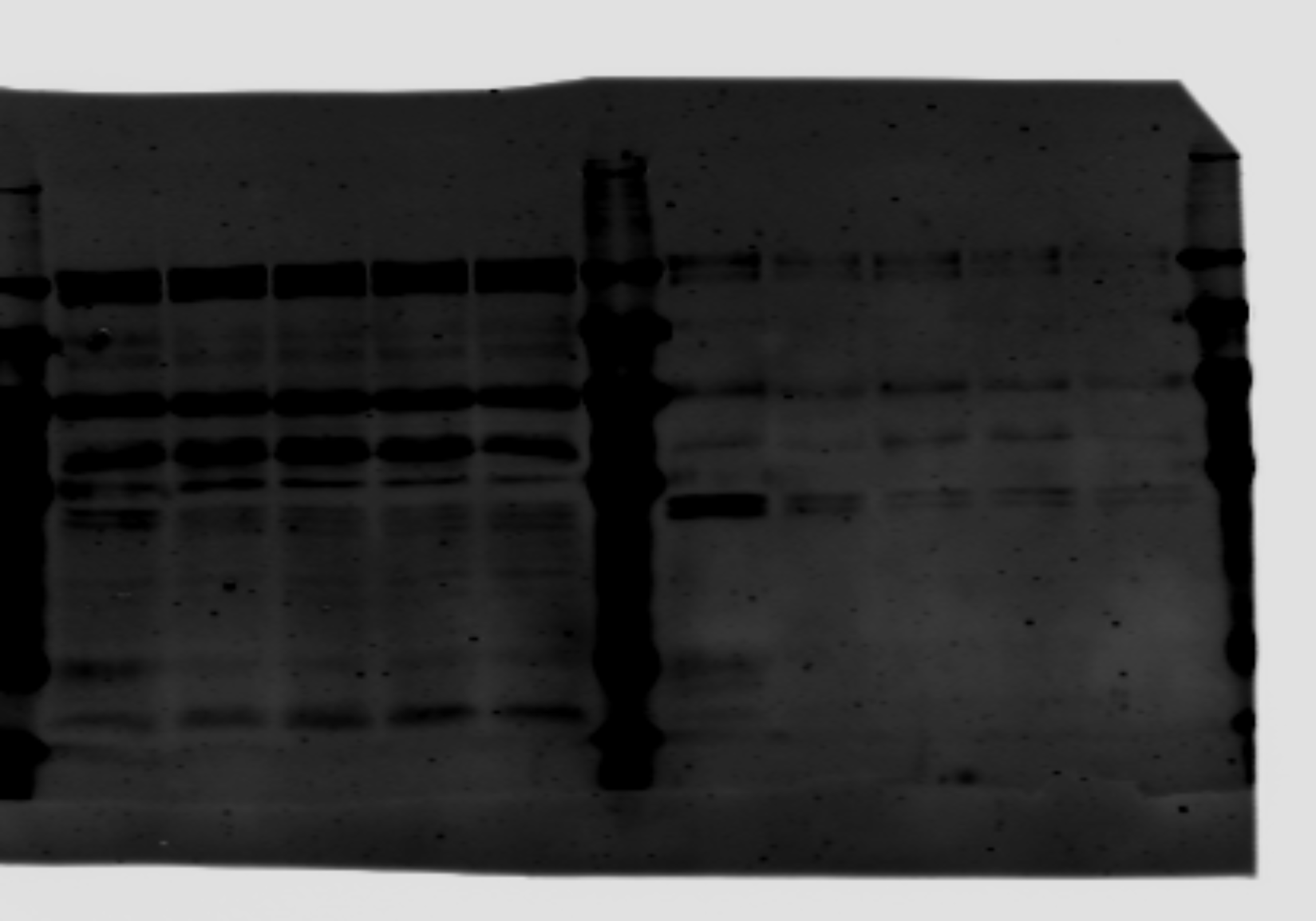


Anti AC-H3 17KD

1

2

3

4

5


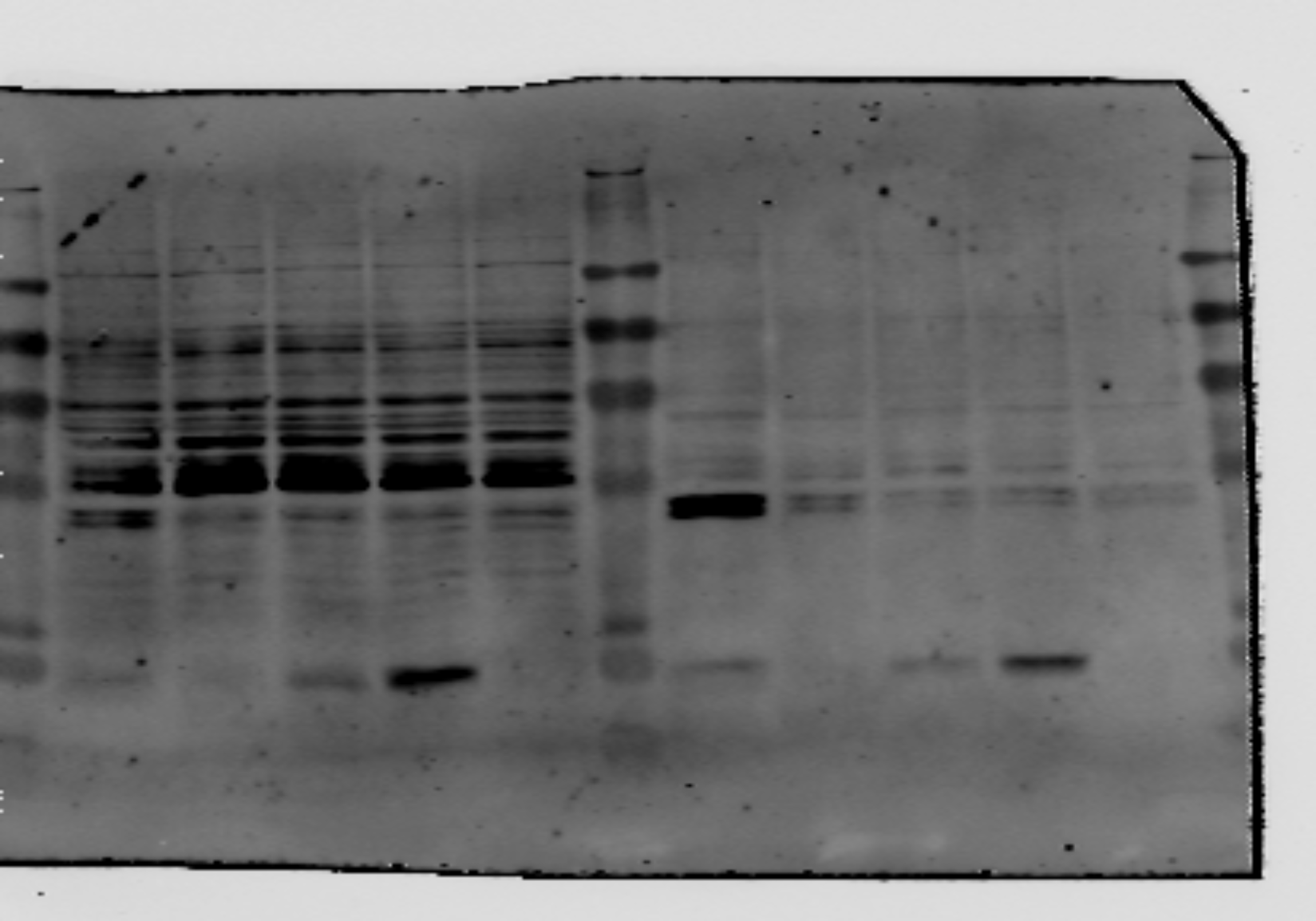


1

2

3

4

5

Anti HP1A 26KD


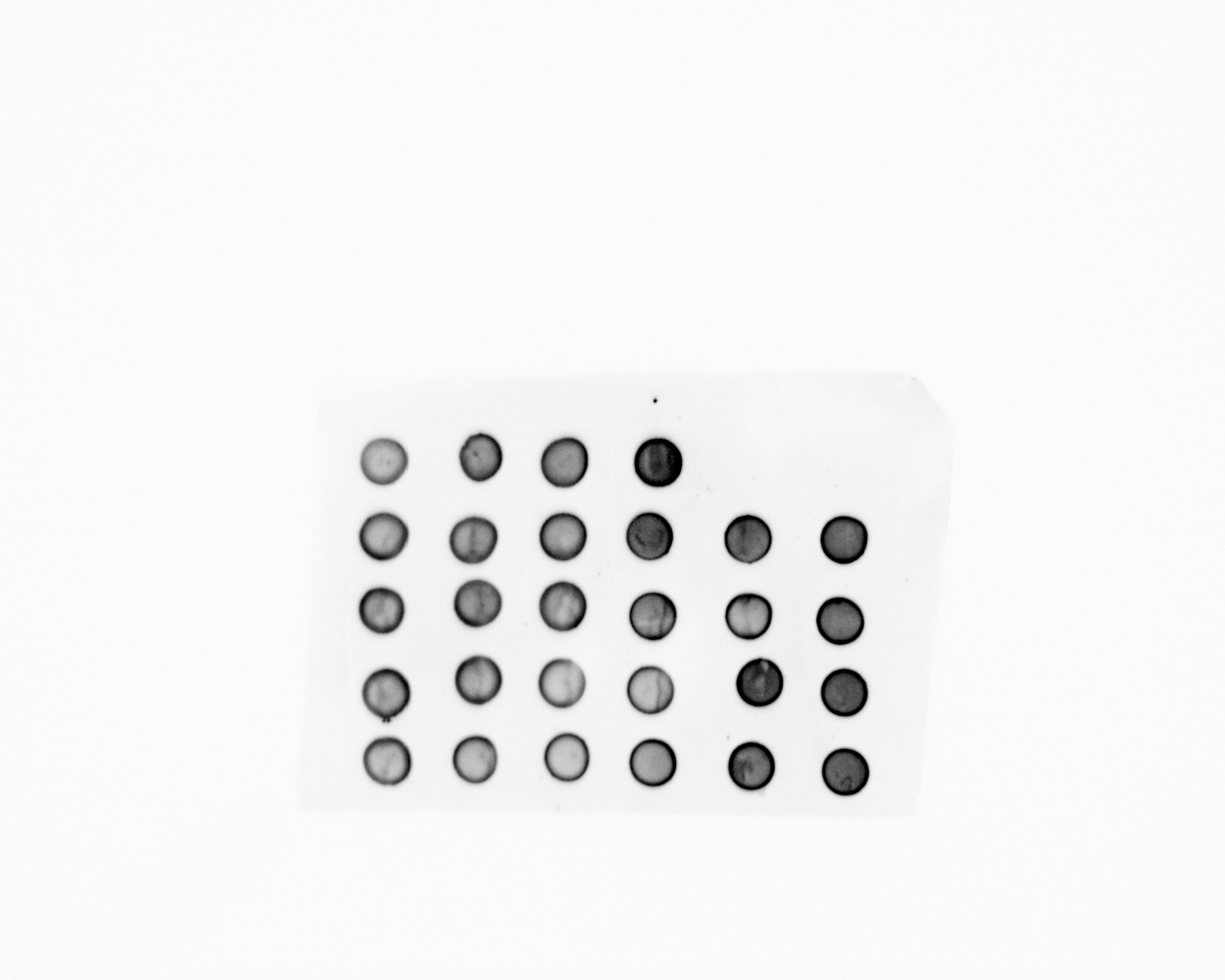


Cisplatin

2DG

SAHA

Rom

Dec


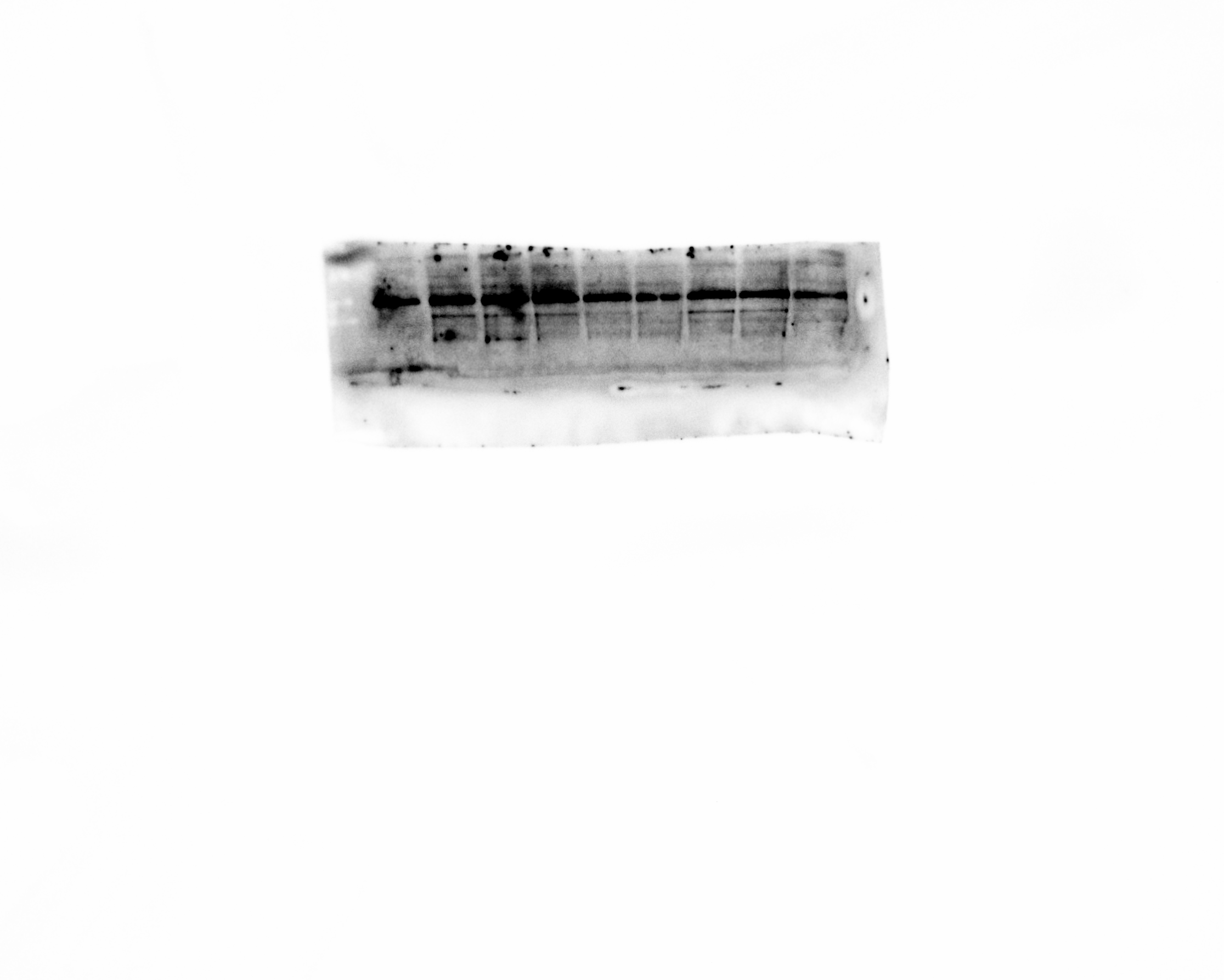


Anti γh2aX 15KD

SKOV3

MCF7

B

A

C

D

**Supplementary Figure 7**

E


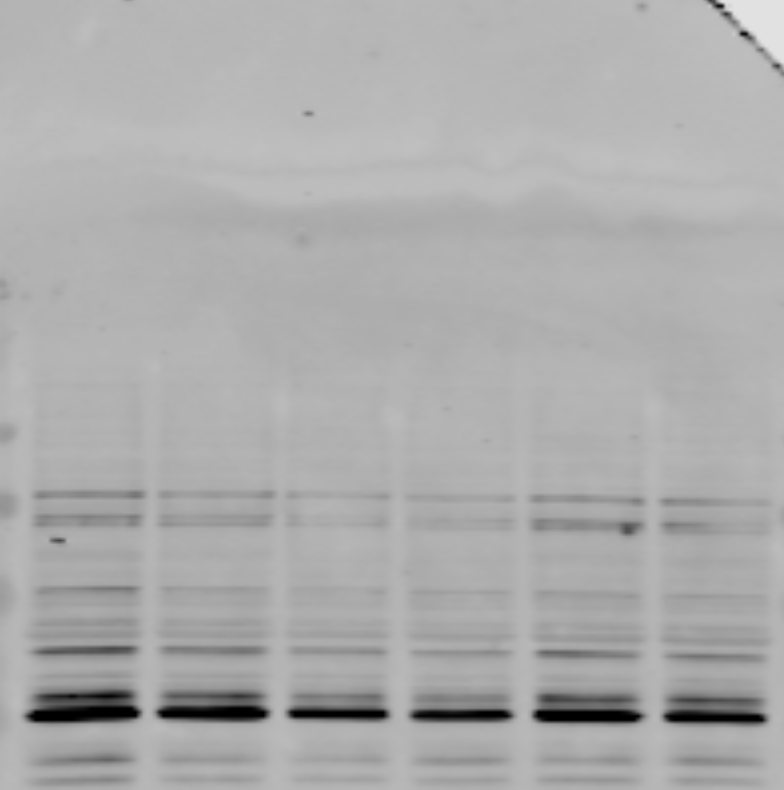


1

3

4

5

2

5

Anti H3 17KD

F

**
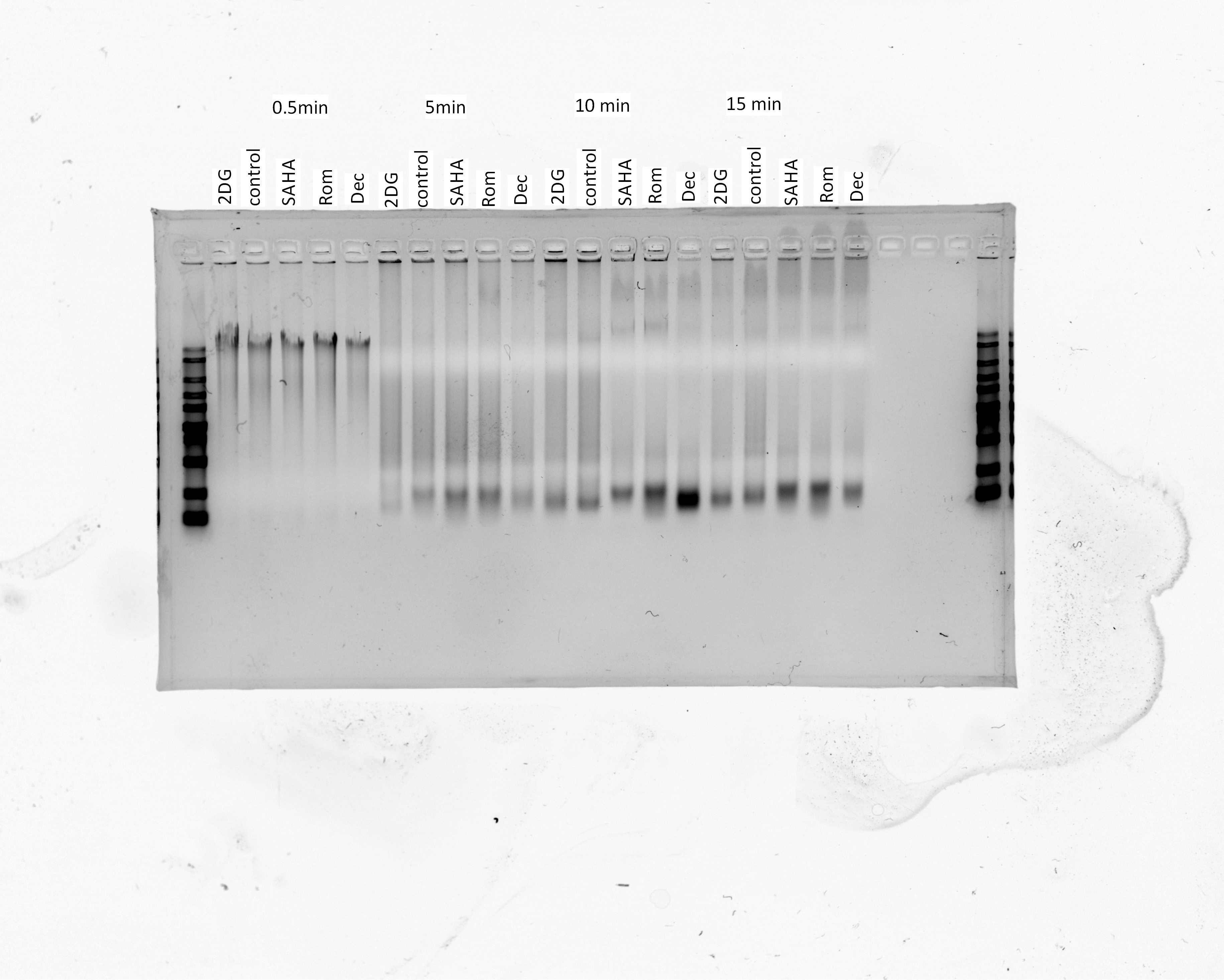
**


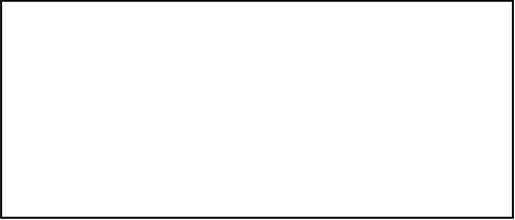

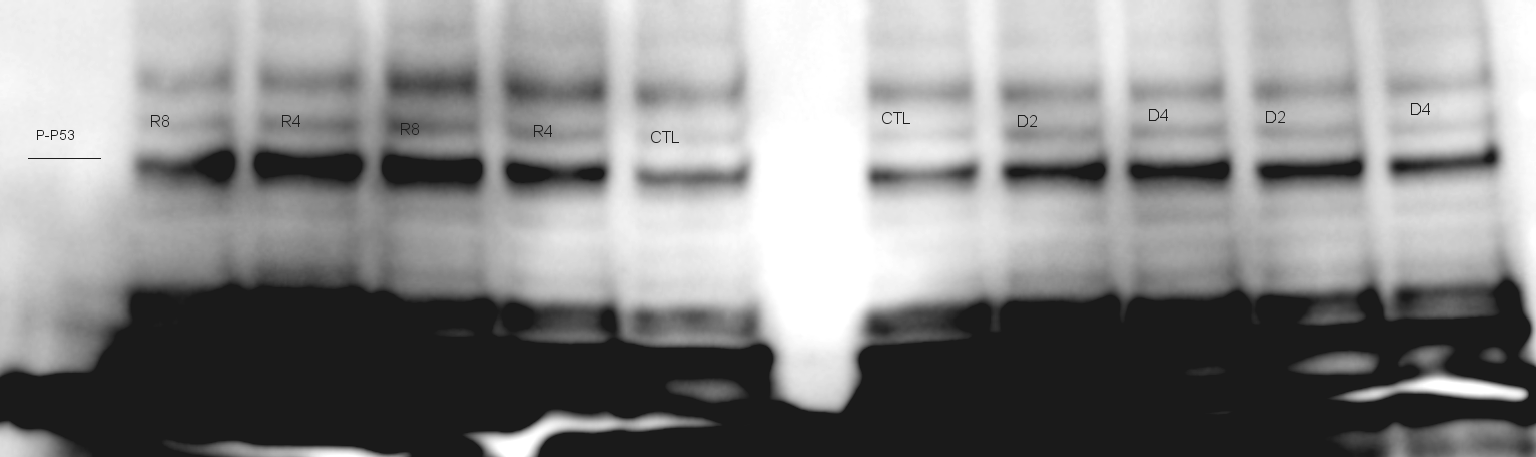

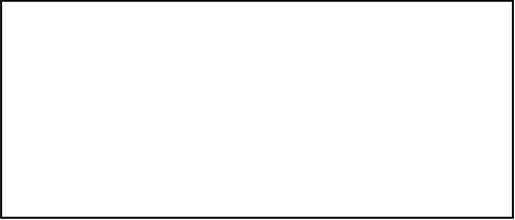

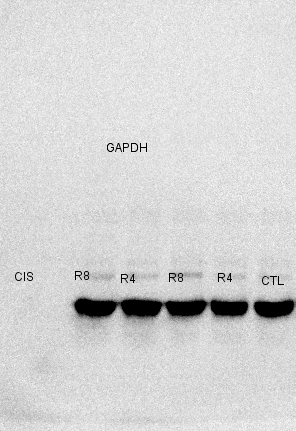

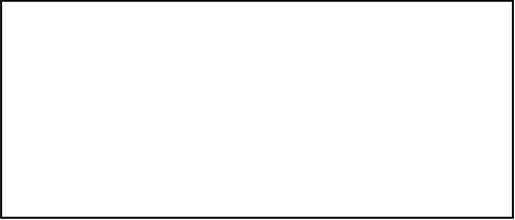

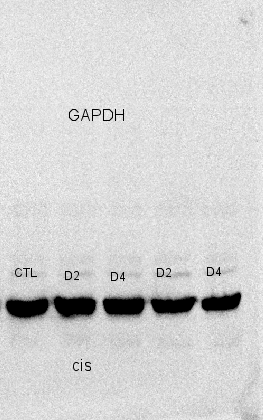

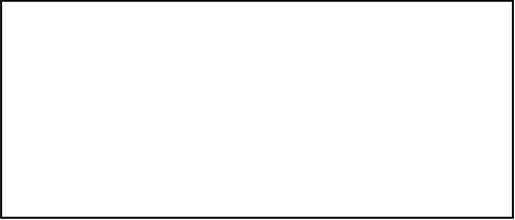

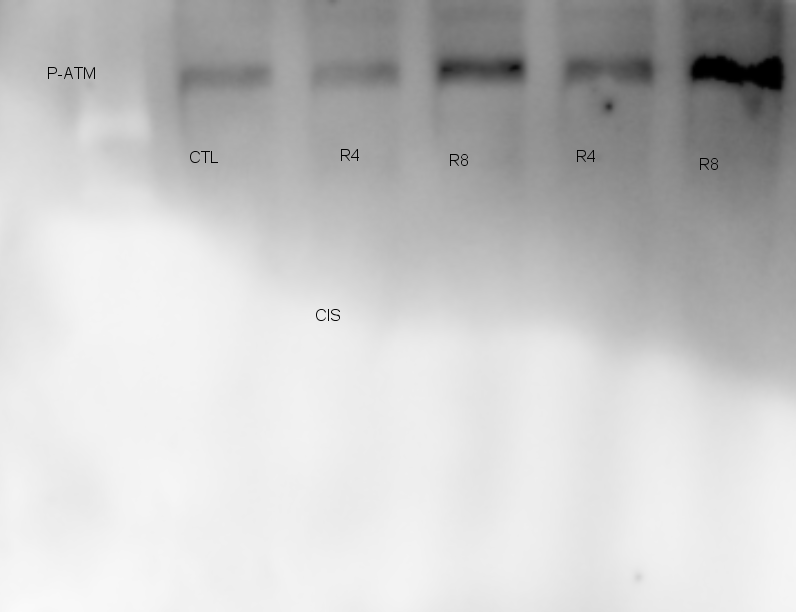

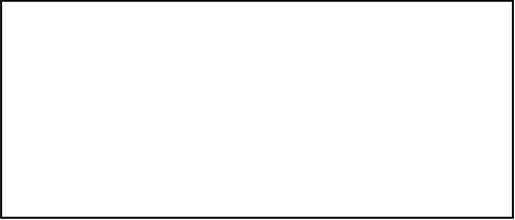

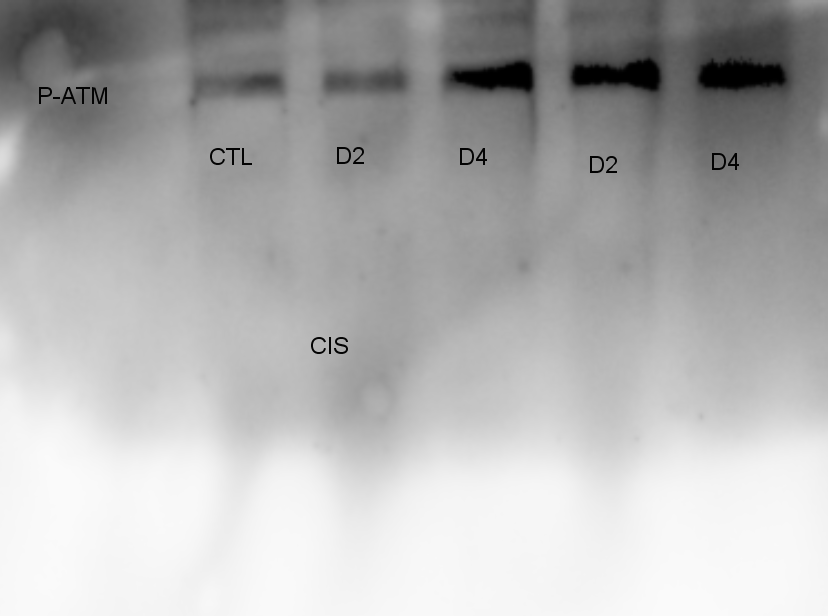


G

H


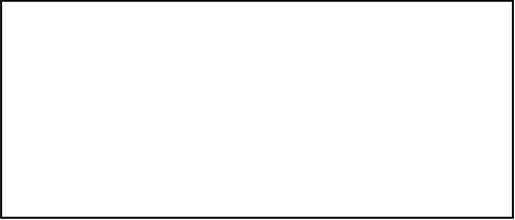

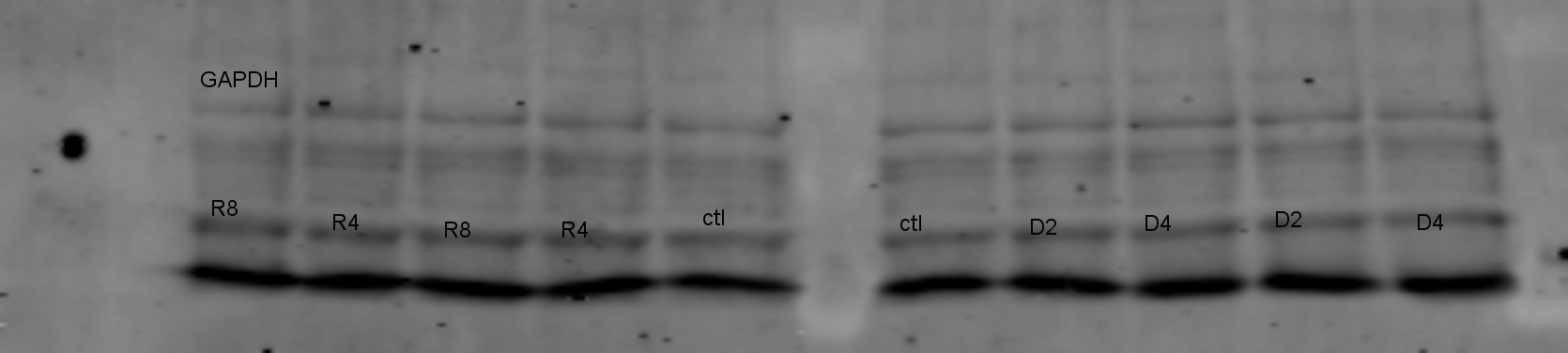

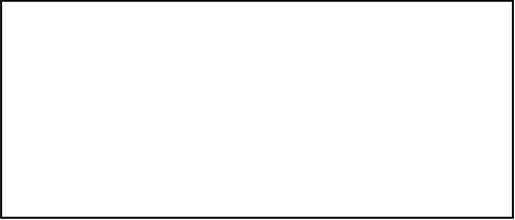

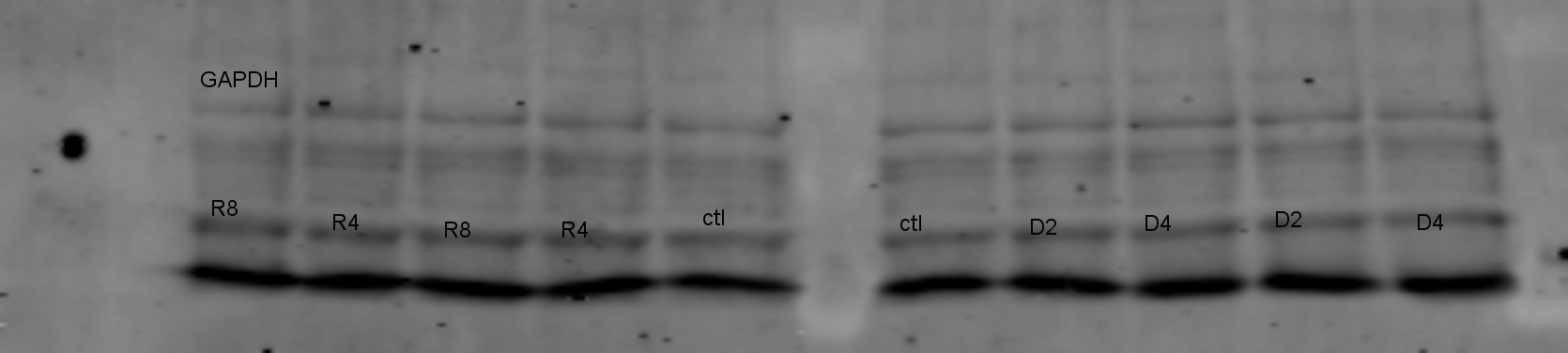

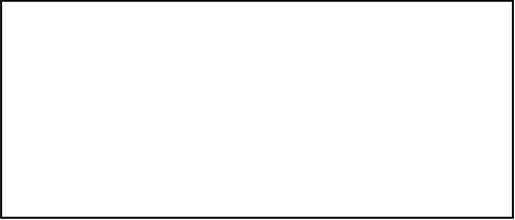

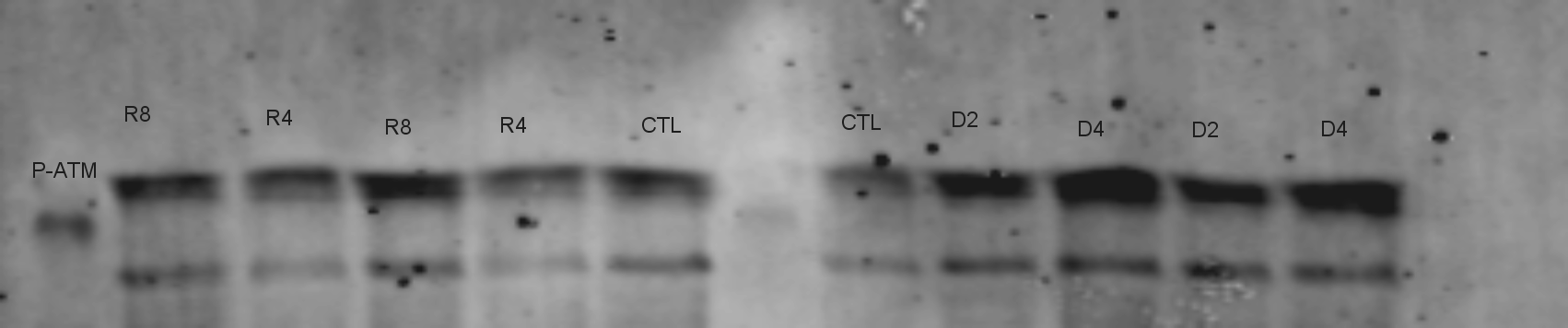

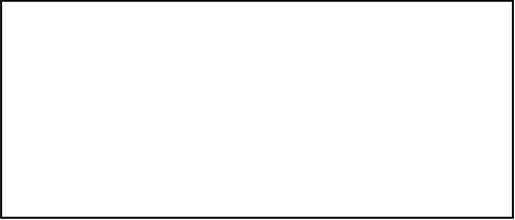

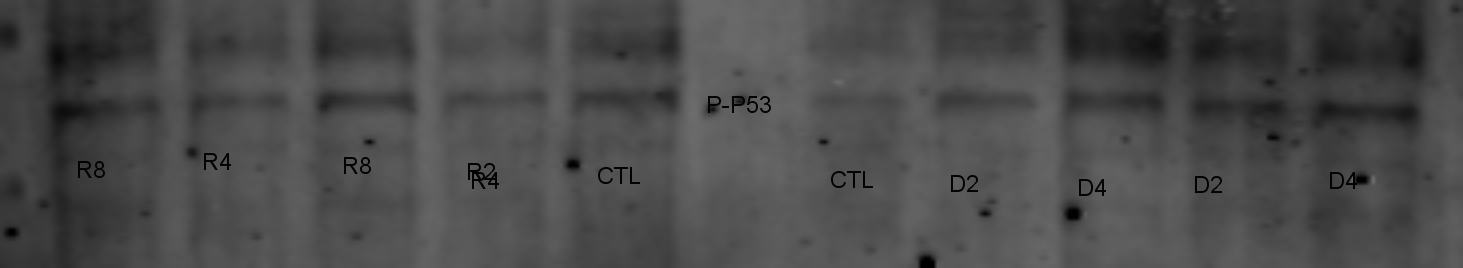


I


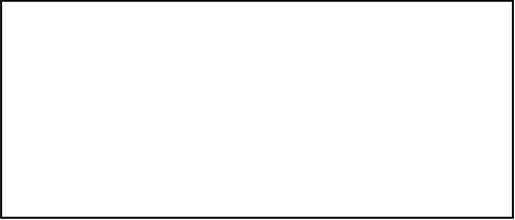

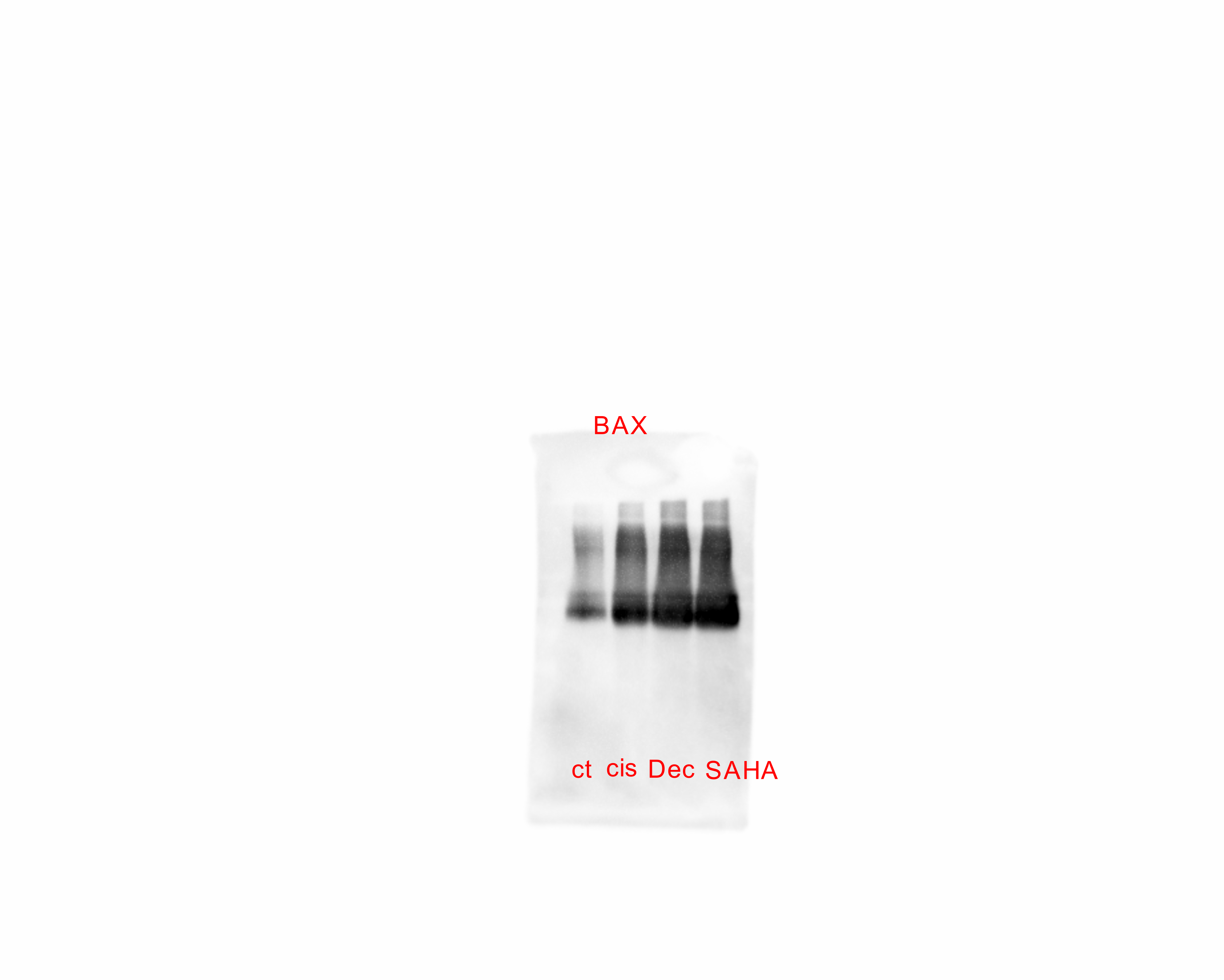

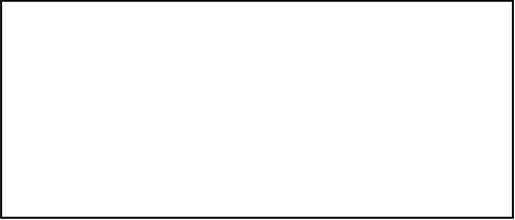

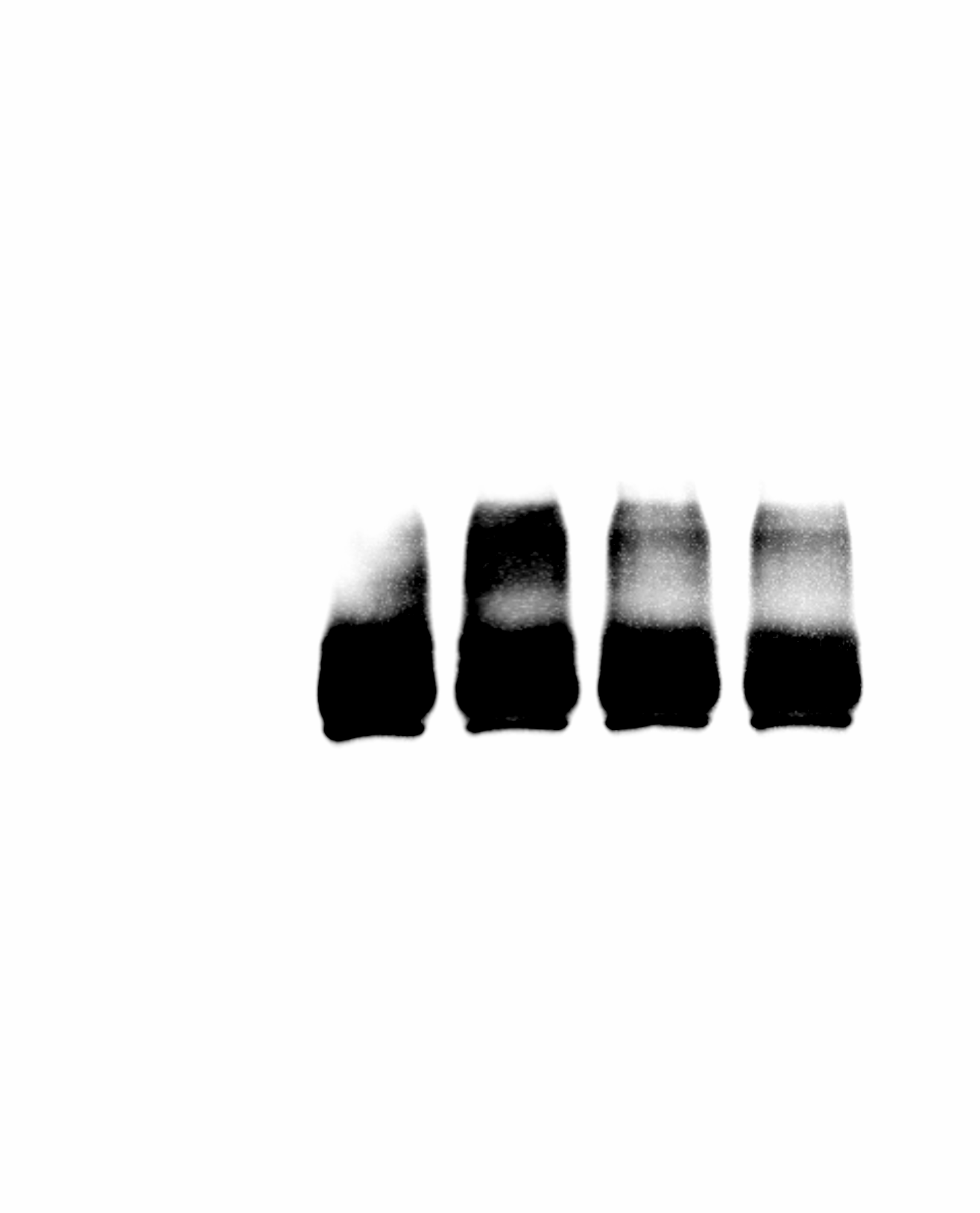


Tublin control cis Dec+cis SAHA+cis


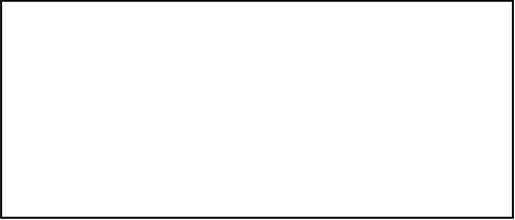

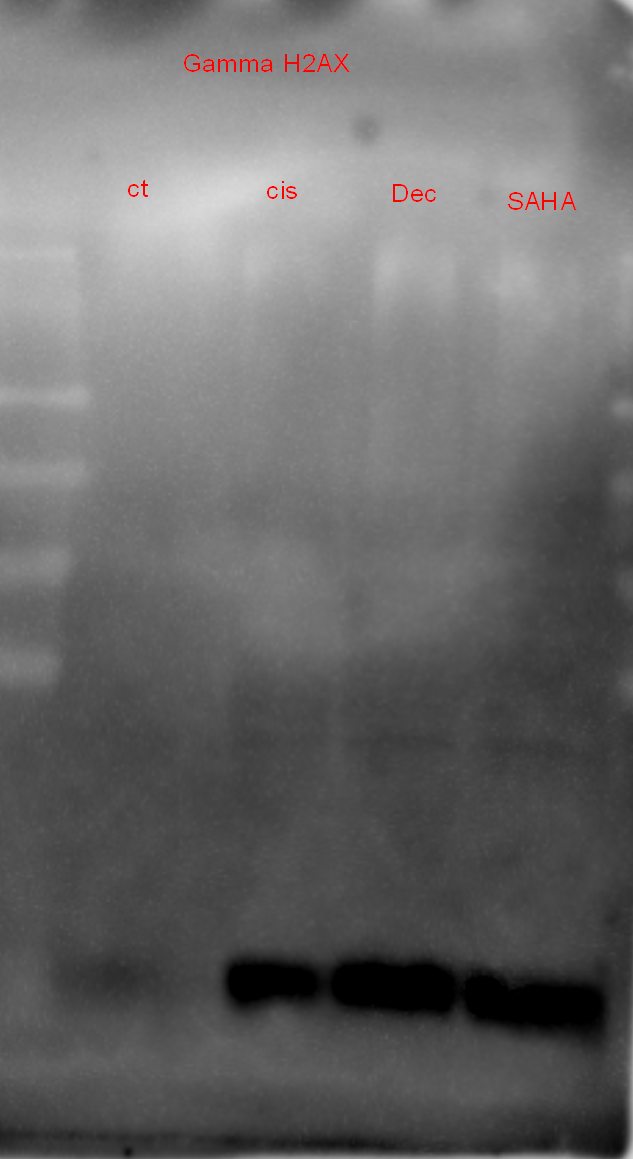

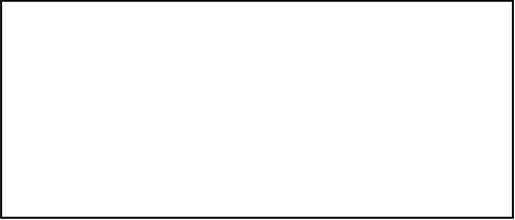

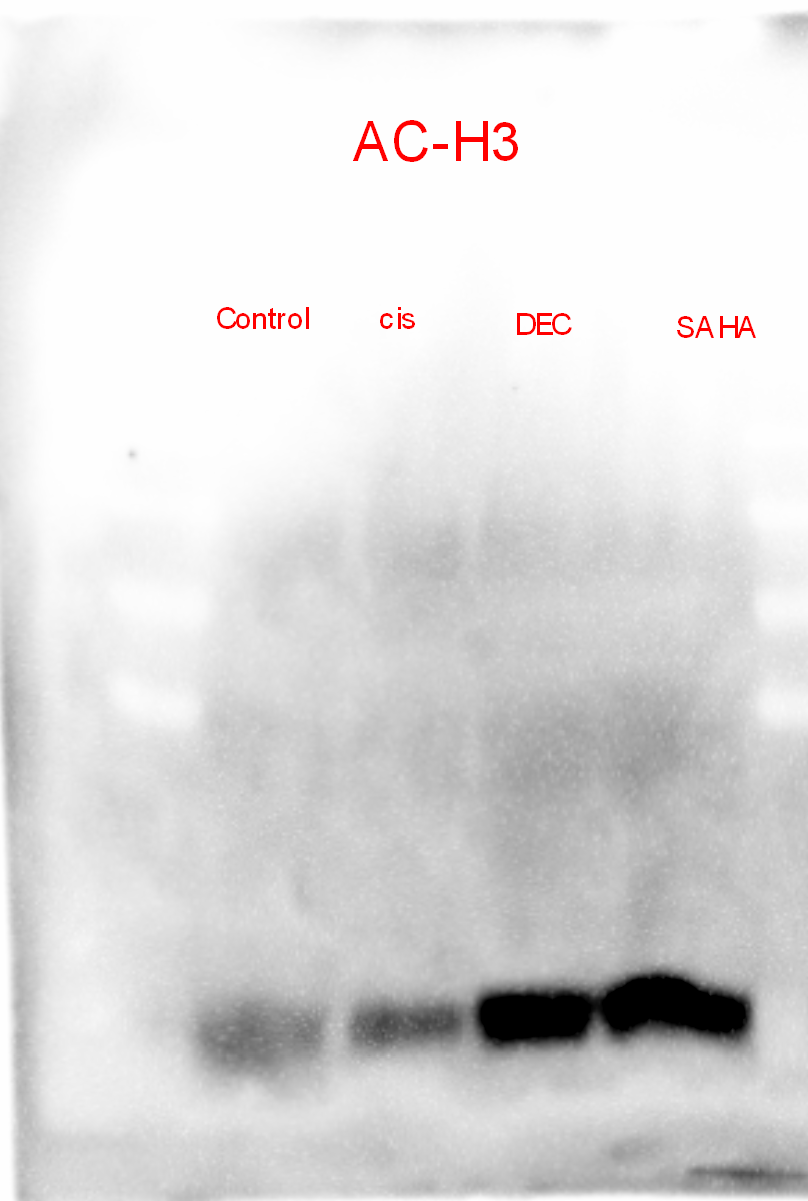

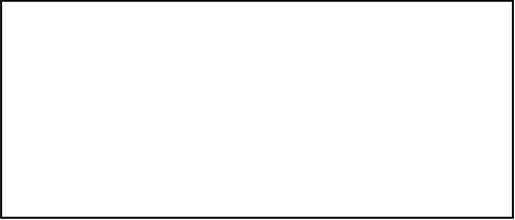

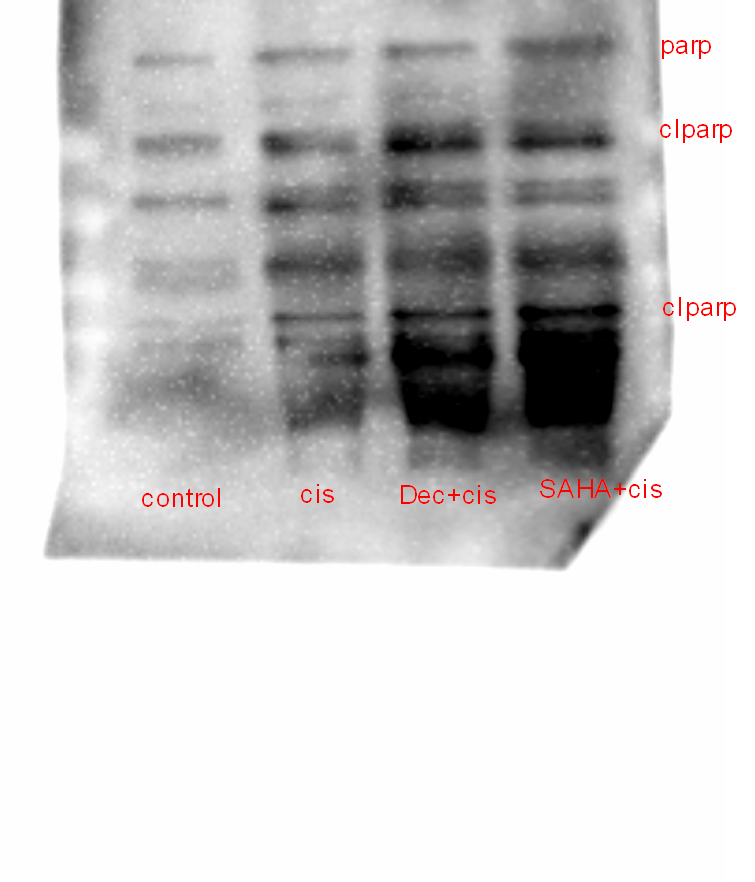

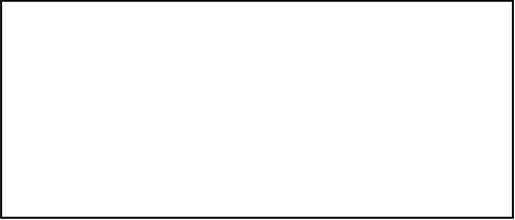

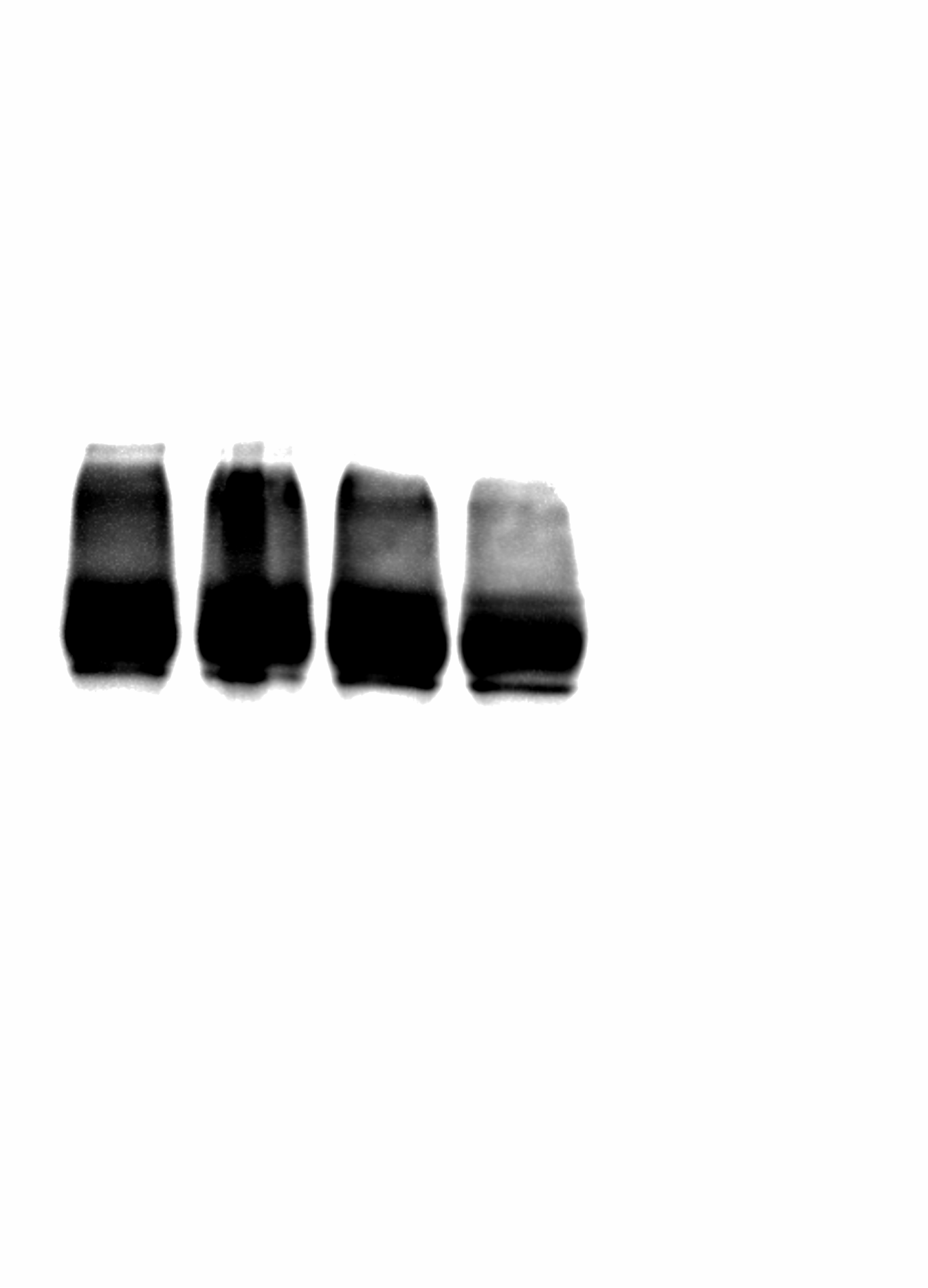


Tublin control IR Dec+IR SAHA+IR


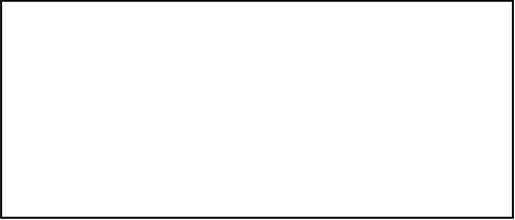

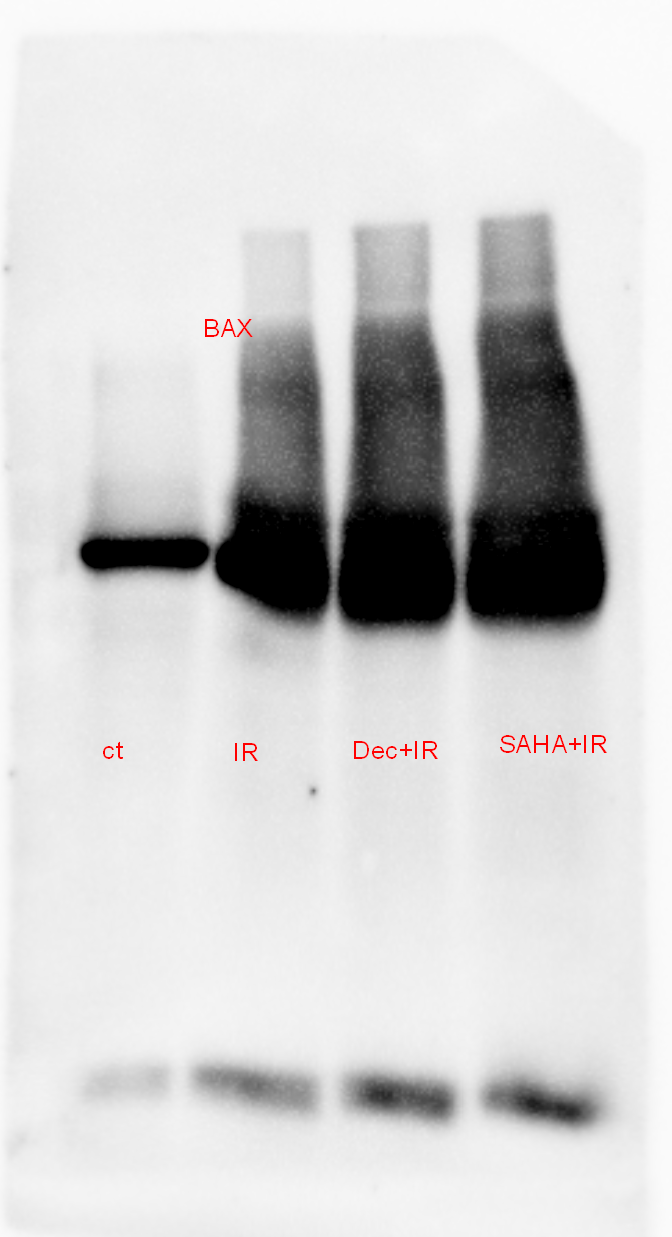

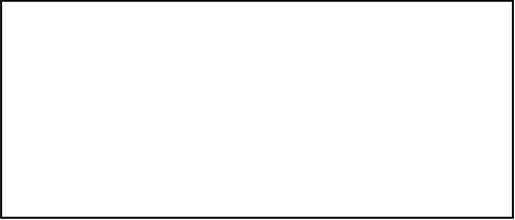

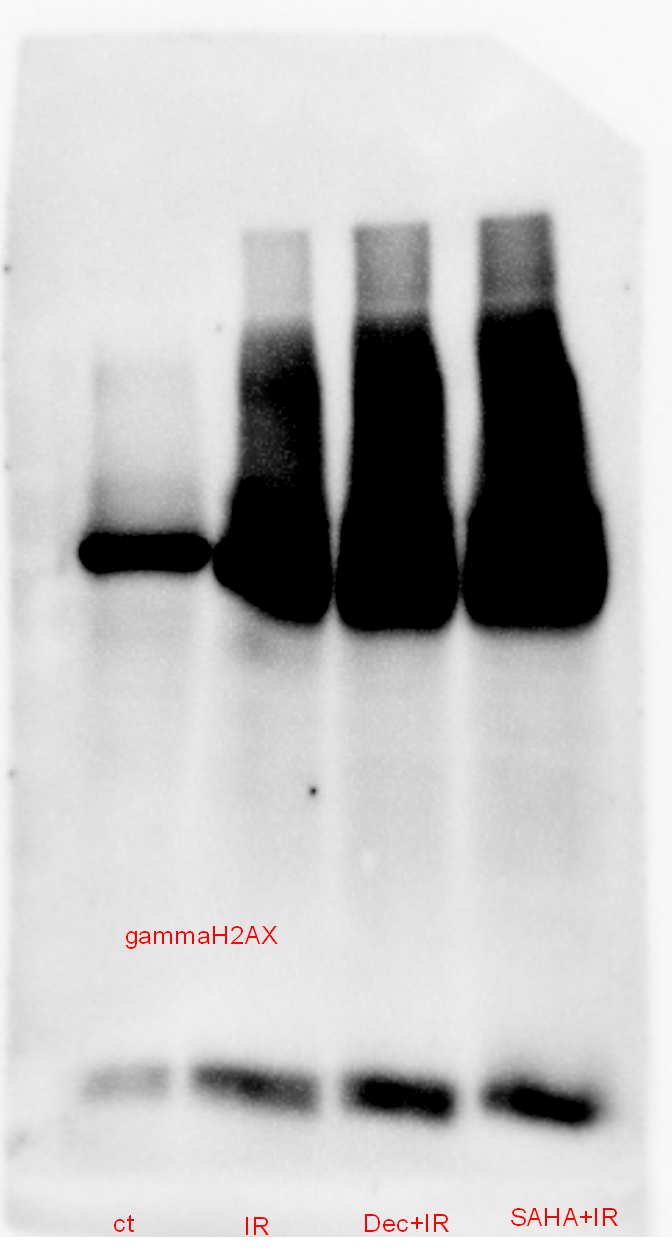

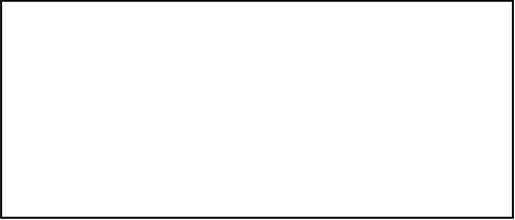

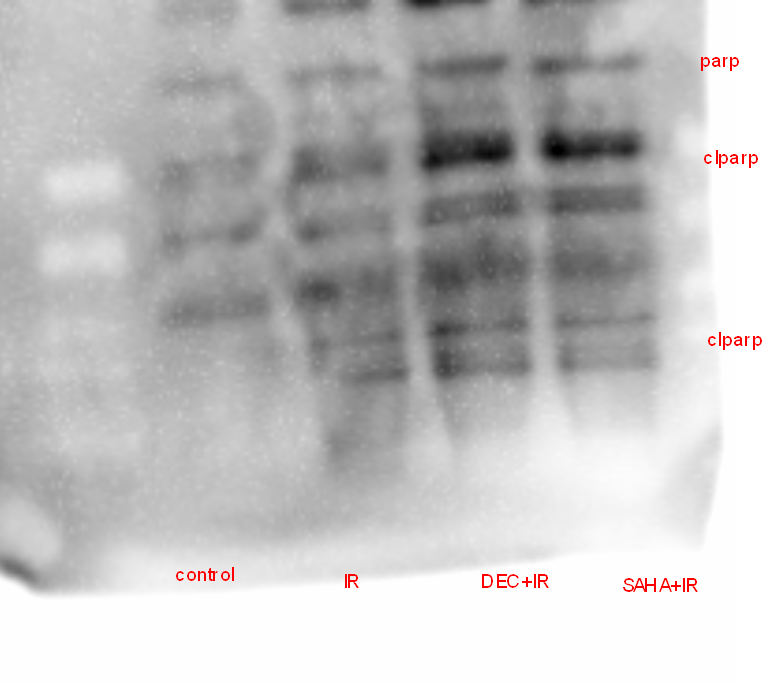


**Supplementary Figure 7**

**Figure S7 Full-length blots/gels figures** Lane 1 is the sample treated with 2-DG; Lane 2 is control; Lane 3 is treated with SAHA; Lane 4 is treated with Rom; Lane 5 is treated Rom. **A, G, H** are full-length blots figures of Figure 3. **B** is full-length blots figures of Figure 4. **C, D, E** are full-length blots figures of Figure 5, **F** is full length gel figure of Figure 5. **I** is full-length blots figures of Figure 6.

**References for Table1**

1. Kaminskas, E., Farrell, A. T., Wang, Y. C., Sridhara, R. & Pazdur, R. FDA drug approval summary: azacitidine (5-azacytidine, Vidaza) for injectable suspension. *Oncologist* **10**, 176-182, doi:10.1634/theoncologist.10-3-176 (2005).
2. Mann, B. S., Johnson, J. R., Cohen, M. H., Justice, R. & Pazdur, R. FDA approval summary: vorinostat for treatment of advanced primary cutaneous T-cell lymphoma. *Oncologist* **12**, 1247-1252, doi:10.1634/theoncologist.12-10-1247 (2007).
3. Kantarjian, H. *et al.* Decitabine improves patient outcomes in myelodysplastic syndromes: results of a phase III randomized study. *Cancer* **106**, 1794-1803, doi:10.1002/cncr.21792 (2006).
4. Piekarz, R. L. *et al.* Phase II multi-institutional trial of the histone deacetylase inhibitor romidepsin as monotherapy for patients with cutaneous T-cell lymphoma. *J Clin Oncol* **27**, 5410-5417, doi:10.1200/JCO.2008.21.6150 (2009).
5. Verstovsek, S. *et al.* A double-blind, placebo-controlled trial of ruxolitinib for myelofibrosis. *N Engl J Med* **366**, 799-807, doi:10.1056/NEJMoa1110557 (2012).
6. O'Connor, O. A. *et al.* Belinostat in Patients With Relapsed or Refractory Peripheral T-Cell Lymphoma: Results of the Pivotal Phase II BELIEF (CLN-19) Study. *J Clin Oncol* **33**, 2492-2499, doi:10.1200/JCO.2014.59.2782 (2015).
7. San-Miguel, J. F. *et al.* Panobinostat plus bortezomib and dexamethasone versus placebo plus bortezomib and dexamethasone in patients with relapsed or relapsed and refractory multiple myeloma: a multicentre, randomised, double-blind phase 3 trial. *Lancet Oncol* **15**, 1195-1206, doi:10.1016/S1470-2045(14)70440-1 (2014).

**References for Table2**

1. Modesitt, S. C., Sill, M., Hoffman, J. S., Bender, D. P. & Gynecologic Oncology, G. A phase II study of vorinostat in the treatment of persistent or recurrent epithelial ovarian or primary peritoneal carcinoma: a Gynecologic Oncology Group study. *Gynecol Oncol* **109**, 182-186, doi:10.1016/j.ygyno.2008.01.009 (2008).
2. Haas, N. B. *et al.* Phase II trial of vorinostat in advanced melanoma. *Invest New Drugs* **32**, 526-534, doi:10.1007/s10637-014-0066-9 (2014).
3. Braiteh, F. *et al.* Phase I study of epigenetic modulation with 5-azacytidine and valproic acid in patients with advanced cancers. *Clin Cancer Res* **14**, 6296-6301, doi:10.1158/1078-0432.CCR-08-1247 (2008).
4. Reid, T. *et al.* Phase II trial of the histone deacetylase inhibitor pivaloyloxymethyl butyrate (Pivanex, AN-9) in advanced non-small cell lung cancer. *Lung Cancer* **45**, 381-386, doi:10.1016/j.lungcan.2004.03.002 (2004).
5. Cassier, P. A. *et al.* A phase II trial of panobinostat in patients with advanced pretreated soft tissue sarcoma. A study from the French Sarcoma Group. *Br J Cancer* **109**, 909-914, doi:10.1038/bjc.2013.442 (2013).
6. Shapiro, G. I. *et al.* The effect of food on the bioavailability of panobinostat, an orally active pan-histone deacetylase inhibitor, in patients with advanced cancer. *Cancer Chemother Pharmacol* **69**, 555-562, doi:10.1007/s00280-011-1758-x (2012).
7. Banerji, U. *et al.* A phase I pharmacokinetic and pharmacodynamic study of CHR-3996, an oral class I selective histone deacetylase inhibitor in refractory solid tumors. *Clin Cancer Res* **18**, 2687-2694, doi:10.1158/1078-0432.CCR-11-3165 (2012).
8. Giaccone, G. *et al.* Phase II study of belinostat in patients with recurrent or refractory advanced thymic epithelial tumors. *J Clin Oncol* **29**, 2052-2059, doi:10.1200/JCO.2010.32.4467 (2011).
9. Venugopal, B. *et al.* A phase I study of quisinostat (JNJ-26481585), an oral hydroxamate histone deacetylase inhibitor with evidence of target modulation and antitumor activity, in patients with advanced solid tumors. *Clin Cancer Res* **19**, 4262-4272, doi:10.1158/1078-0432.CCR-13-0312 (2013).
10. Yeo, W. *et al.* Epigenetic therapy using belinostat for patients with unresectable hepatocellular carcinoma: a multicenter phase I/II study with biomarker and pharmacokinetic analysis of tumors from patients in the Mayo Phase II Consortium and the Cancer Therapeutics Research Group. *J Clin Oncol* **30**, 3361-3367, doi:10.1200/JCO.2011.41.2395 (2012).
11. Prakash, S. *et al.* Chronic oral administration of CI-994: a phase 1 study. *Invest New Drugs* **19**, 1-11 (2001).
12. Galanis, E. *et al.* Phase II trial of vorinostat in recurrent glioblastoma multiforme: a north central cancer treatment group study. *J Clin Oncol* **27**, 2052-2058, doi:10.1200/JCO.2008.19.0694 (2009).
13. Krug, L. M. *et al.* Potential role of histone deacetylase inhibitors in mesothelioma: clinical experience with suberoylanilide hydroxamic acid. *Clin Lung Cancer* **7**, 257-261, doi:10.3816/CLC.2006.n.003 (2006).
14. Yong, W. P. *et al.* Phase I and pharmacodynamic study of an orally administered novel inhibitor of histone deacetylases, SB939, in patients with refractory solid malignancies. *Ann Oncol* **22**, 2516-2522, doi:10.1093/annonc/mdq784 (2011).
15. Krug, L. M. *et al.* Vorinostat in patients with advanced malignant pleural mesothelioma who have progressed on previous chemotherapy (VANTAGE-014): a phase 3, double-blind, randomised, placebo-controlled trial. *Lancet Oncol* **16**, 447-456, doi:10.1016/S1470-2045(15)70056-2 (2015).
16. Stearns, V. *et al.* Biomarker modulation following short-term vorinostat in women with newly diagnosed primary breast cancer. *Clin Cancer Res* **19**, 4008-4016, doi:10.1158/1078-0432.CCR-13-0033 (2013).
17. Fujiwara, Y. *et al.* Phase I and pharmacokinetic study of vorinostat (suberoylanilide hydroxamic acid) in Japanese patients with solid tumors. *Cancer Sci* **100**, 1728-1734, doi:10.1111/j.1349-7006.2009.01237.x (2009).
18. Doi, T. *et al.* Evaluation of safety, pharmacokinetics, and efficacy of vorinostat, a histone deacetylase inhibitor, in the treatment of gastrointestinal (GI) cancer in a phase I clinical trial. *Int J Clin Oncol* **18**, 87-95, doi:10.1007/s10147-011-0348-6 (2013).
19. Woyach, J. A. *et al.* Lack of therapeutic effect of the histone deacetylase inhibitor vorinostat in patients with metastatic radioiodine-refractory thyroid carcinoma. *J Clin Endocrinol Metab* **94**, 164-170, doi:10.1210/jc.2008-1631 (2009).
20. Traynor, A. M. *et al.* Vorinostat (NSC# 701852) in patients with relapsed non-small cell lung cancer: a Wisconsin Oncology Network phase II study. *J Thorac Oncol* **4**, 522-526 (2009).
21. Luu, T. H. *et al.* A phase II trial of vorinostat (suberoylanilide hydroxamic acid) in metastatic breast cancer: a California Cancer Consortium study. *Clin Cancer Res* **14**, 7138-7142, doi:10.1158/1078-0432.CCR-08-0122 (2008).
22. Brunetto, A. T. *et al.* First-in-human, pharmacokinetic and pharmacodynamic phase I study of Resminostat, an oral histone deacetylase inhibitor, in patients with advanced solid tumors. *Clin Cancer Res* **19**, 5494-5504, doi:10.1158/1078-0432.CCR-13-0735 (2013).
23. Haigentz, M., Jr. *et al.* Phase II trial of the histone deacetylase inhibitor romidepsin in patients with recurrent/metastatic head and neck cancer. *Oral Oncol* **48**, 1281-1288, doi:10.1016/j.oraloncology.2012.05.024 (2012).
24. Mohammed, T. A. *et al.* A pilot phase II study of valproic acid for treatment of low-grade neuroendocrine carcinoma. *Oncologist* **16**, 835-843, doi:10.1634/theoncologist.2011-0031 (2011).
25. Mackay, H. J. *et al.* Phase II trial of the histone deacetylase inhibitor belinostat in women with platinum resistant epithelial ovarian cancer and micropapillary (LMP) ovarian tumours. *Eur J Cancer* **46**, 1573-1579, doi:10.1016/j.ejca.2010.02.047 (2010).
26. Juergens, R. A. *et al.* Combination epigenetic therapy has efficacy in patients with refractory advanced non-small cell lung cancer. *Cancer Discov* **1**, 598-607, doi:10.1158/2159-8290.CD-11-0214 (2011).
27. Atmaca, A. *et al.* Valproic acid (VPA) in patients with refractory advanced cancer: a dose escalating phase I clinical trial. *Br J Cancer* **97**, 177-182, doi:10.1038/sj.bjc.6603851 (2007).
28. Ramalingam, S. S. *et al.* Phase II study of belinostat (PXD101), a histone deacetylase inhibitor, for second line therapy of advanced malignant pleural mesothelioma. *J Thorac Oncol* **4**, 97-101, doi:10.1097/JTO.0b013e318191520c (2009).
29. Chu, B. F. *et al.* Phase I study of 5-aza-2'-deoxycytidine in combination with valproic acid in non-small-cell lung cancer. *Cancer Chemother Pharmacol* **71**, 115-121, doi:10.1007/s00280-012-1986-8 (2013).
30. Blumenschein, G. R., Jr. *et al.* Phase II trial of the histone deacetylase inhibitor vorinostat (Zolinza, suberoylanilide hydroxamic acid, SAHA) in patients with recurrent and/or metastatic head and neck cancer. *Invest New Drugs* **26**, 81-87, doi:10.1007/s10637-007-9075-2 (2008).
31. Vansteenkiste, J. *et al.* Early phase II trial of oral vorinostat in relapsed or refractory breast, colorectal, or non-small cell lung cancer. *Invest New Drugs* **26**, 483-488, doi:10.1007/s10637-008-9131-6 (2008).
32. Amiri-Kordestani, L. *et al.* Phase I trial of a new schedule of romidepsin in patients with advanced cancers. *Clin Cancer Res* **19**, 4499-4507, doi:10.1158/1078-0432.CCR-13-0095 (2013).
33. Razak, A. R. *et al.* Phase I clinical, pharmacokinetic and pharmacodynamic study of SB939, an oral histone deacetylase (HDAC) inhibitor, in patients with advanced solid tumours. *Br J Cancer* **104**, 756-762, doi:10.1038/bjc.2011.13 (2011).
